# A theoretical model of the West Nile Virus survival data

**DOI:** 10.1186/s12865-017-0206-z

**Published:** 2017-06-21

**Authors:** James K. Peterson, Alison M. Kesson, Nicholas J. C. King

**Affiliations:** 1 Department of Biological Sciences, Department of Mathematical Sciences, Clemson University, Martin Hall O-304, BOX 340975, Clemson, SC, 340975 USA; 2 Discipline of Child and Adolescent Health and the Marie Bashir Institute for Emerging Infectious Diseases and Biosecurity, University of Sydney, Sydney, NSW Australia; 3 Sydney Children’s Hospital Network, Westmead, Sydney, NSW Australia; 4 Discipline of Pathology, University of Sydney, Sydney, NSW Australia

**Keywords:** Auto-immune, West Nile Virus, Decoy model, MHC-I upregulation, IFN- *γ*, Immunopathology

## Abstract

**Background:**

In this work, we develop a theoretical model that explains the survival data in West Nile Virus infection.

**Results:**

We build a model based on three cell populations in an infected host; the collateral damage cells, the infected dividing cell, and the infected non-dividing cells. T cell-mediated lysis of each of these populations is dependent on the level of MHC-1 upregulation, which is different in the two infected cell populations, interferon-gamma and free virus levels.

**Conclusions:**

The model allows us to plot a measure of host health versus time for a range of initial viral doses and from that infer the dependence of minimal health versus viral dose. This inferred functional relationship between the minimal host health and viral dose is very similar to the data that has been collected for WNV survival curves under experimental conditions.

## Background

Viruses in the family Flaviviridae are single-stranded, plus-sense RNA viruses, principally transmitted by mosquitoes and ticks. They are either viscerotropic, causing diseases such as dengue and yellow fever, or neurotropic, causing central nervous system disease, like West Nile virus encephalitis, Japanese encephalitis or Saint Louis encephalitis, all of which may be fatal. These viruses are found worldwide and the diseases they cause pose a significant public health burden. A careful discussion of the many facets of flavivirus infections can be found in [1], and we therefore confine our introductory remarks to the features salient to this paper.

Virus-infected cells process virus proteins into peptide fragments in the proteasome and these are bound to class I major histocompatibility complex molecules (MHC-I) in the endoplasmic reticulum. With the transport of MHC molecules to the cell surface, viral peptides displayed in the context of MHC on the infected cell can be recognized by virus-specific cytotoxic T lymphocytes (CTL). Such CTL kill infected cells by a variety of lytic effector molecules prior to the release of mature virus progeny from infected cells, thereby progressively reducing virus numbers and ultimately eradicating virus from surviving hosts (reviewed in [2]).

Paradoxically, flaviviruses such as West Nile (WNV) and others, directly induce increased expression of MHC-I, as well as MHC-II, and several adhesion molecules involved in immune recognition by CTL. This increased expression results in a marked increase in the efficiency of recognition and killing of infected cells by WNV-specific CTL ([3, 4]), because although the affinity of individual T cell receptor (TcR)-MHC-virus peptide interaction is unchanged, the multiple intermolecular interactions increases the avidity of interaction of virus-specific CTL with the infected cell. This increased avidity also enables the functional interaction with MHC ^*hi*^, infected cells by CTL clones of low MHC-virus peptide affinity, i.e., clones previously below the recognition threshold. Some of these low-affinity CTL clones are likely to be self-reactive [4] or even able to recognize MHC without peptide specificity [5]. Thus, the increased avidity brought about by high MHC expression enables low-affinity, self-reactive clones, not normally involved in anti-viral immune responses, to lyse both infected and uninfected target cells [6]. Adhesion molecules such as ICAM-1, as non-specific accessory molecules upregulated by WNV infection, also increase the avidity of CTL-target cell interactions, further lowering the affinity threshold for T cell recognition and target cell lysis [7]. In addition, interferon- *γ* (IFN- *γ*), released by CTL on recognition of their cognate ligand, strongly increases MHC and ICAM-1 expression on neighboring target cells, further contributing to the progressive increase in avidity of interaction between CTL and target cells [8]. In this context, the stage of the cell cycle in which a cell is infected is also important; cells infected by WNV in *G*_0_ (resting) increase MHC-I expression by 6-10-fold, compared to a 2-3-fold increase observed in cells infected during the cell cycle (*G*_1_, S, *G*_2_+*M*) [3]. WNV-infected *G*_0_ cells are approximately 10-fold more susceptible to CTL lysis than infected cycling cells exposed to the same CTL [3]. Thus, WNV-infected cycling cells are less easily recognized, while the avidity of interaction between CTL and infected *G*_0_ cells is significantly enhanced by the higher levels of MHC-I and ICAM-1. Notably, WNV replicates significantly better in the poorly recognized cycling cells than in *G*_0_ cells. In vivo, most cells are in *G*_0_, presumably presenting an easy CTL target once infected, but a small population of productively infected cycling cells maintaining a low immunological profile, could substantially increase the probability of virus transmission to the next host [4]. As indicated above, the release of IFN- *γ* upon target cell recognition by CTL would also increase MHC and ICAM-1 expression on uninfected cells in the vicinity of virus- infected cells, making high MHC-I-expressing (uninfected) cells a potential target for lysis by low affinity virus-specific and/or self-reactive (i.e., cross-reactive) CTL clones. The collateral destruction of uninfected cells by low-affinity clones of this kind would cause substantial additional damage to the brain, with corresponding increases in morbidity and mortality [9]. We have developed a model of the collateral damage caused by a West Nile Virus infection, which is supported by simulation results [10]. A discussion of the underlying simulation code can be found in [11]. We have also used these simulation results to explain the unusual ragged survival data seen in West Nile Virus infections [12]. The previous work, in focusing on very low level details, based on first principles, could be regarded as a *micro* model. Here we develop a *macro* model, based on a much higher level approach and ideas. Each model has its pros and cons but we believe each brings complementary insights into a very complicated problem. Our focus here is therefore on developing a *macro* level theoretical model of how the survival of a host depends on the level of initial viral dose. We believe this approach provides an abstract focus which can also be directed towards more general models of immunopathology and we will briefly mention those connections at the end. These more general models of autoimmune response are the focus of additional work we are doing.

## WNV survival data

**Fig. 1:**
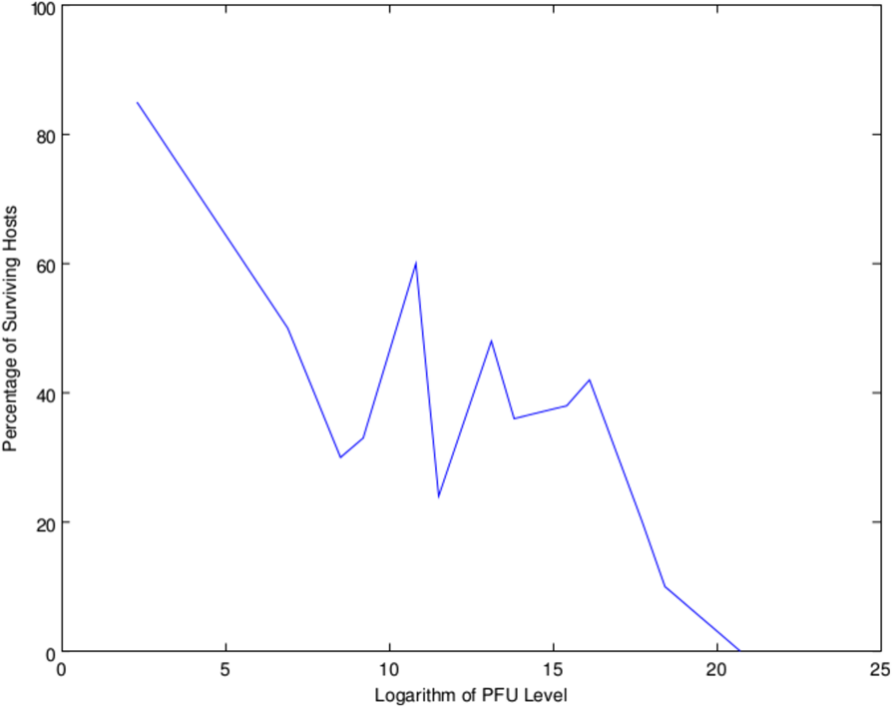
Simulated survival curve

In a survival experiment, a population of animals such as mice are infected with a common amount of virus and the number of animals that die are counted. This experiment is repeated for a range of virus. With most viruses, the survival experiment gives a classical dose-response curve which progressively and smoothly decays down to a survival of 0 animals at high virus. However, WNV has a peculiar survival curve as shown in [12] but we can easily simulate what such a curve looks like as we have done in Fig. 1. The purpose of this paper is to find a theoretical model that explains this data. The derivations here use standard ideas from advanced calculus and differential equations. The necessary background can be found in any nonlinear modeling text such as [13]. We assume we have a large population of cells ***T*** which consists of cells which are dividing and are infected, ***D***, cells which are not dividing but are infected, ***N***, non infected cells, ***H*** and non infected cells which will be removed due to auto immune action which we call ***C***, for collateral damage. We assume all of these variables depend on the three parameters, ***I***, the IFN- *γ* signal; the MHCI upregulation factor ***U*** and the free virus level ***A***. The approach we take here is a common one in such a nonlinear interaction environment. An example of how this is used to develop a model of diabetes detection can be found in [13].

## The ***CDN*** model

We assume the dynamics here are

$$\begin{array}{@{}rcl@{}} \boldsymbol{C}^{\prime}(t) &=& F_{1}(\boldsymbol{C}, \boldsymbol{D}, \boldsymbol{N})\\ \boldsymbol{D}^{\prime}(t) &=& F_{2}(\boldsymbol{C}, \boldsymbol{D}, \boldsymbol{N})\\ \boldsymbol{N}^{\prime}(t) &=& F_{3}(\boldsymbol{C}, \boldsymbol{D}, \boldsymbol{N}) \end{array} $$

There are then three nonlinear interaction functions *F*_1_, *F*_2_ and *F*_3_ because we know ***C***, ***D*** and ***N*** depend on each other’s levels in very complicated ways. Usually, we assume the initial dose ***S***_***0***_ gives rise to some fraction of infected cells which will differ in both the dividing and nondividing cells. In our previous WNV simulations of collateral damage [10], we assume *p*_0_=1.0*e*−3 of the initial dose gives rise to infected cells. This gives the number of infected cells to be *p*_0_ ***S***_***0***_ which is split into *p*_1_ *p*_0_ ***S***_***0***_ infected non dividing cells - i.e. in ***N***, and *p*_2_ *p*_0_ ***S***_***0***_ dividing infected cells, i.e. in ***D***, where *p*_1_+*p*_2_=1. Typically, we let *p*_1_=0.99 and *p*_2_=0.01 but other choices could be made. Thus, the total amount of virus that goes into infected cells is *p*_0_ ***S***_***0***_ and the amount of free virus is therefore (1−*p*_0_) ***S***_***0***_. Thus, we could expect ***C***_***0***_=0, ***D***_***0***_=*p*_2_ *p*_0_ ***S***_***0***_ and ***N***_***0***_=***D***_***0***_=*p*_1_ *p*_0_ ***S***_***0***_. However, we will explicitly assume we are starting from a point of equilibrium prior to the administration of the viral dose ***S***_***0***_. We could assume there is always some level of collateral damage, ***C***_***0***_ in a host, but we will not do that. We will therefore assume ***C***, ***D*** and ***N*** have achieved these values ***C***_***0***_=0, ***D***_***0***_=0 and ***N***_***0***_=0 right before the moment of infection. Hence, we don’t expect there to be initial contribution to ***C*** ^′^(0), ***D*** ^′^(0) and ***N*** ^′^(0); i.e. *F*_1_(***C***_***0***_,***D***_***0***_,***N***_***0***_)=0, *F*_2_(***C***_***0***_,***D***_***0***_,***N***_***0***_)=0 and *F*_3_(***C***_***0***_,***D***_***0***_,***N***_***0***_)=0. We are interested in the deviation of ***C***, ***D*** and ***N*** from their optimal values ***C***_***0***_, ***D***_***0***_ and ***N***_***0***_, so let ***c***=***C***−***C***_***0***_, ***d***=***D***−***D***_***0***_ and ***n***=***N***−***N***_***0***_. We can then write ***C***=***C***_***0***_+***c***, ***D***=***D***_***0***_+***d*** and ***N***=***N***_***0***_+***n*** The model can then be rewritten as

$$\begin{array}{@{}rcl@{}} (\boldsymbol{C_{0}} + \boldsymbol{c})^{\prime}(t) &=& F_{1}(\boldsymbol{C_{0}} + \boldsymbol{c},\boldsymbol{D_{0}} + \boldsymbol{d}, \boldsymbol{N_{0}} + \boldsymbol{n}) \\ (\boldsymbol{D_{0}} + \boldsymbol{d})^{\prime}(t) &=& F_{2}((\boldsymbol{C_{0}} + \boldsymbol{c},\boldsymbol{D_{0}} + \boldsymbol{d}, \boldsymbol{N_{0}} + \boldsymbol{n})\\ (\boldsymbol{D_{0}} + \boldsymbol{d})^{\prime}(t) &=& F_{3}((\boldsymbol{C_{0}} + \boldsymbol{c},\boldsymbol{D_{0}} + \boldsymbol{d}, \boldsymbol{N_{0}} + \boldsymbol{n}) \end{array} $$

or

$$\begin{array}{@{}rcl@{}} \boldsymbol{c}^{\prime}(t) &=& F_{1}(\boldsymbol{C_{0}} + \boldsymbol{c},\boldsymbol{D_{0}} + \boldsymbol{d},,\boldsymbol{N_{0}} + \boldsymbol{n}) \\ \boldsymbol{d}^{\prime}(t) &=& F_{2}((\boldsymbol{C_{0}} + \boldsymbol{c},\boldsymbol{D_{0}} + \boldsymbol{d}, \boldsymbol{N_{0}} + \boldsymbol{n})\\ \boldsymbol{n}^{\prime}(t) &=& F_{3}((\boldsymbol{C_{0}} + \boldsymbol{c},\boldsymbol{D_{0}} + \boldsymbol{d}, \boldsymbol{N_{0}} + \boldsymbol{n}) \end{array} $$

Next, we do a standard tangent plane approximation on the nonlinear dynamics functions *F*_1_, *F*_2_ and *F*_3_ to derive approximation dynamics.

### Linearization details

To develop a useful approximation to the ***CDN*** dynamics, we use an appropriate tangent plane approximation on the nonlinear dynamics functions *F*_1_, *F*_2_ and *F*_3_. The mathematics behind this approximation come from multivariate calculus and can easily be reviewed if required. The standard tangent plane expansions is as follows.

$$\begin{array}{@{}rcl@{}} F_{1}(\boldsymbol{C_{0}} &+& \boldsymbol{c},\boldsymbol{D_{0}} + \boldsymbol{d}, \boldsymbol{N_{0}} + \boldsymbol{n}) = F_{1}(\boldsymbol{C_{0}},\boldsymbol{D_{0}},\boldsymbol{N_{0}})\\ &+& \frac{\partial F_{1}}{\partial \boldsymbol{c}}(\boldsymbol{C_{0}}, \boldsymbol{D_{0}},\boldsymbol{D_{0}}) \: \boldsymbol{c}\\ &+& \frac{\partial F_{1}}{\partial \boldsymbol{d}}(\boldsymbol{C_{0}}, \boldsymbol{D_{0}},\boldsymbol{D_{0}}) \: \boldsymbol{d}+ \frac{\partial F_{1}}{\partial \boldsymbol{d}}(\boldsymbol{C_{0}}, \boldsymbol{D_{0}},\boldsymbol{D_{0}}) \: \boldsymbol{n} + E_{1}^{F} \end{array} $$

$$\begin{array}{@{}rcl@{}} F_{2}(\boldsymbol{C_{0}} &+& \boldsymbol{c},\boldsymbol{D_{0}} + \boldsymbol{d}, \boldsymbol{N_{0}} + \boldsymbol{n}) = F_{2}(\boldsymbol{C_{0}},\boldsymbol{D_{0}},\boldsymbol{N_{0}})\\ &+& \frac{\partial F_{2}}{\partial \boldsymbol{c}}(\boldsymbol{C_{0}}, \boldsymbol{D_{0}},\boldsymbol{D_{0}}) \: \boldsymbol{c}\\ &+& \frac{\partial F_{2}}{\partial \boldsymbol{d}}(\boldsymbol{C_{0}}, \boldsymbol{D_{0}},\boldsymbol{D_{0}}) \: \boldsymbol{d}+ \frac{\partial F_{2}}{\partial \boldsymbol{d}}(\boldsymbol{C_{0}}, \boldsymbol{D_{0}},\boldsymbol{D_{0}}) \: \boldsymbol{n} +E_{2}^{F}\\ F_{3}(\boldsymbol{C_{0}} &+& \boldsymbol{c},\boldsymbol{D_{0}} + \boldsymbol{d}, \boldsymbol{N_{0}} + \boldsymbol{n}) = F_{3}(\boldsymbol{C_{0}},\boldsymbol{D_{0}},\boldsymbol{N_{0}})\\ &+& \frac{\partial F_{3}}{\partial \boldsymbol{c}}(\boldsymbol{C_{0}}, \boldsymbol{D_{0}},\boldsymbol{D_{0}}) \: \boldsymbol{c}\\ &+& \frac{\partial F_{3}}{\partial \boldsymbol{d}}(\boldsymbol{C_{0}}, \boldsymbol{D_{0}},\boldsymbol{D_{0}}) \: \boldsymbol{d}+ \frac{\partial F_{3}}{\partial \boldsymbol{d}}(\boldsymbol{C_{0}}, \boldsymbol{D_{0}},\boldsymbol{D_{0}}) \: \boldsymbol{n} +E_{3}^{F} \end{array} $$

But the terms *F*_1_(***C***_***0***_,***D***_***0***_,***N***_***0***_)=0, *F*_2_(***C***_***0***_,***D***_***0***_,***N***_***0***_)=0 and *F*_3_(***C***_***0***_,***D***_***0***_,***N***_***0***_)=0. so we can simplify to

$$\begin{array}{@{}rcl@{}} F_{1}(\boldsymbol{C_{0}} &+& \boldsymbol{c},\boldsymbol{D_{0}} + \boldsymbol{d}, \boldsymbol{N_{0}} + \boldsymbol{n}) = \frac{\partial F_{1}}{\partial \boldsymbol{c}}(\boldsymbol{C_{0}}, \boldsymbol{D_{0}},\boldsymbol{D_{0}}) \: \boldsymbol{c}\\ &+& \frac{\partial F_{1}}{\partial \boldsymbol{d}}(\boldsymbol{C_{0}}, \boldsymbol{D_{0}},\boldsymbol{D_{0}}) \: \boldsymbol{d}\\ &+& \frac{\partial F_{1}}{\partial \boldsymbol{d}}(\boldsymbol{C_{0}}, \boldsymbol{D_{0}},\boldsymbol{D_{0}}) \: \boldsymbol{n}+ E_{1}^{F}\\ F_{2}(\boldsymbol{C_{0}} &+& \boldsymbol{c},\boldsymbol{D_{0}} + \boldsymbol{d}, \boldsymbol{N_{0}} + \boldsymbol{n}) = \frac{\partial F_{2}}{\partial \boldsymbol{c}}(\boldsymbol{C_{0}}, \boldsymbol{D_{0}},\boldsymbol{D_{0}}) \: \boldsymbol{c}\\ &+& \frac{\partial F_{2}}{\partial \boldsymbol{d}}(\boldsymbol{C_{0}}, \boldsymbol{D_{0}},\boldsymbol{D_{0}}) \: \boldsymbol{d}\\ &+&\frac{\partial F_{2}}{\partial \boldsymbol{d}}(\boldsymbol{C_{0}}, \boldsymbol{D_{0}},\boldsymbol{D_{0}}) \: \boldsymbol{n} +E_{2}^{F}\\ F_{3}(\boldsymbol{C_{0}} &+& \boldsymbol{c},\boldsymbol{D_{0}} + \boldsymbol{d}, \boldsymbol{N_{0}} + \boldsymbol{n}) = \frac{\partial F_{3}}{\partial \boldsymbol{c}}(\boldsymbol{C_{0}}, \boldsymbol{D_{0}},\boldsymbol{D_{0}}) \: \boldsymbol{c}\\ &+& \frac{\partial F_{3}}{\partial \boldsymbol{d}}(\boldsymbol{C_{0}}, \boldsymbol{D_{0}},\boldsymbol{D_{0}}) \: \boldsymbol{d}+ \frac{\partial F_{3}}{\partial \boldsymbol{d}}(\boldsymbol{C_{0}}, \boldsymbol{D_{0}},\boldsymbol{D_{0}}) \: \boldsymbol{n}+E_{3}^{F} \end{array} $$

It seems reasonable to assume that since we are so close to ordinary operating conditions, the errors $E_{1}^{F}$, $E_{2}^{F}$ and $E_{3}^{F}$ will be negligible. Also, to save space, we let ()^*o*^ denote that we are evaluating the partial derivatives at the point (***C***_***0***_,***D***_***0***_,***D***_***0***_). Thus our model approximation is

$$\begin{array}{@{}rcl@{}} F_{1}(\boldsymbol{C_{0}} + \boldsymbol{c},\boldsymbol{D_{0}} + \boldsymbol{d}, \boldsymbol{N_{0}} + \boldsymbol{n}) &\approx& \left(\frac{\partial F_{1}}{\partial \boldsymbol{c}}\right)^{o} \boldsymbol{c}+ \left(\frac{\partial F_{1}}{\partial \boldsymbol{d}}\right)^{o} \boldsymbol{d}\\ &&+ \left(\frac{\partial F_{1}}{\partial \boldsymbol{n}}\right)^{o} \boldsymbol{n}\\ F_{2}(\boldsymbol{C_{0}} + \boldsymbol{c},\boldsymbol{D_{0}} + \boldsymbol{d}, \boldsymbol{N_{0}} + \boldsymbol{n}) &\approx& \left(\frac{\partial F_{2}}{\partial \boldsymbol{c}}\right)^{o} \boldsymbol{c}+ \left(\frac{\partial F_{2}}{\partial \boldsymbol{d}}\right)^{o} \boldsymbol{d}\\&&+ \left(\frac{\partial F_{2}}{\partial \boldsymbol{n}}\right)^{o} \boldsymbol{n}\\ F_{3}(\boldsymbol{C_{0}} + \boldsymbol{c},\boldsymbol{D_{0}} + \boldsymbol{d}, \boldsymbol{N_{0}} + \boldsymbol{n}) &\approx& \left(\frac{\partial F_{3}}{\partial \boldsymbol{c}}\right)^{o} \boldsymbol{c}+ \left(\frac{\partial F_{3}}{\partial \boldsymbol{d}}\right)^{o} \boldsymbol{d}\\ &&+\left(\frac{\partial F_{3}}{\partial \boldsymbol{n}}\right)^{o} \boldsymbol{n} \end{array} $$

and our corresponding nonlinear dynamics approximation is

$$\begin{array}{@{}rcl@{}} \boldsymbol{c}^{\prime} &\approx& \left(\frac{\partial F_{1}}{\partial \boldsymbol{c}}\right)^{o} \boldsymbol{c}+ \left(\frac{\partial F_{1}}{\partial \boldsymbol{d}}\right)^{o} \boldsymbol{d}+ \left(\frac{\partial F_{1}}{\partial \boldsymbol{n}}\right)^{o} \boldsymbol{n}\\ \boldsymbol{d}^{\prime} &\approx& \left(\frac{\partial F_{2}}{\partial \boldsymbol{c}}\right)^{o} \boldsymbol{c}+ \left(\frac{\partial F_{2}}{\partial \boldsymbol{d}}\right)^{o} \boldsymbol{d}+ \left(\frac{\partial F_{2}}{\partial \boldsymbol{n}}\right)^{o} \boldsymbol{n}\\ \boldsymbol{n}^{\prime} &\approx& \left(\frac{\partial F_{3}}{\partial \boldsymbol{c}}\right)^{o} \boldsymbol{c}+ \left(\frac{\partial F_{3}}{\partial \boldsymbol{d}}\right)^{o} \boldsymbol{d}+ \left(\frac{\partial F_{3}}{\partial \boldsymbol{n}}\right)^{o} \boldsymbol{n} \end{array} $$

We can write this approximation In matrix - vector form also:

$$\begin{array}{@{}rcl@{}} \left[\begin{array}{l} \boldsymbol{c}^{\prime}\\ \boldsymbol{d}^{\prime}\\ \boldsymbol{n}^{\prime} \end{array}\right] &\approx&\left[\begin{array}{lll} F_{1\boldsymbol{c}}^{o} & F_{1\boldsymbol{d}}^{o} & F_{1\boldsymbol{n}}^{o}\\ F_{2\boldsymbol{c}}^{o} & F_{2\boldsymbol{d}}^{o} & F_{2\boldsymbol{n}}^{o}\\ F_{3\boldsymbol{c}}^{o} & F_{3\boldsymbol{d}}^{o} & F_{3\boldsymbol{n}}^{o} \end{array}\right] \left[\begin{array}{l} \boldsymbol{c}\\ \boldsymbol{d}\\ \boldsymbol{n} \end{array}\right] \end{array} $$

where we now use a standard subscript scheme to indicate the partials. Now let’s add IFN- *γ*, upregulation and virus level.

## The CDN IFN- *γ*, upregulation and virus model

We can think each variable ***C***, ***D*** and ***N*** as depending on the interferon level ***I***, the upregulation level ***U*** and the free virus level ***A***. Thus, we have

$$\begin{array}{@{}rcl@{}} F_{1}(\boldsymbol{C}(\boldsymbol{I,U,A}), \boldsymbol{D}(\boldsymbol{I,U,A}), \boldsymbol{N}(\boldsymbol{I,U,A})) &=& H_{1}(\boldsymbol{I,U,A})\\ F_{2}(\boldsymbol{C}(\boldsymbol{I,U,A}), \boldsymbol{D}(\boldsymbol{I,U,A}), \boldsymbol{N}(\boldsymbol{I,U,A})) &=& H_{2}(\boldsymbol{I,U,A})\\ F_{3}(\boldsymbol{C}(\boldsymbol{I,U,A}), \boldsymbol{D}(\boldsymbol{I,U,A}), \boldsymbol{N}(\boldsymbol{I,U,A})) &=& H_{3}(\boldsymbol{I,U,A}) \end{array} $$

We assume the dynamics here are then

$$\begin{array}{@{}rcl@{}} \boldsymbol{C}^{\prime} &=& H_{1}(\boldsymbol{I,U,A}) \\ \boldsymbol{D}^{\prime}&=& H_{2}(\boldsymbol{I,U,A}) \\ \boldsymbol{N}^{\prime}&=& H_{3}(\boldsymbol{I,U,A}) \end{array} $$

As before assume ***C***, ***D*** and ***C*** have achieved the same optimal values ***C***_***0***_=0, ***D***_***0***_=0 and ***N***_***0***_=0 prior to the moment of infection with virus dose ***S***_***0***_. These correspond to the starting values of prior to infection for base values ***I***_***0***_, ***U***_***0***_ and ***A***_***0***_. Initially, we don’t expect IFN- *γ* signals so ***I***_***0***_=0. Eventually, we do expect some level of upregulation due to this initial dose and from experimental data, we expect the upregulation to be proportional to the level of the dose ***S***_***0***_; we will assume this is a simple scaling factor, i.e. ***U***_***0***_=*q*_1_ ***S***_***0***_ for some suitable parameter *q*_1_. Also, once the viral dose is administered, we would expect some fraction of it to remain as free virus which as discussed in [10] is modeled as ***A***_***0***_=(1−*p*_0_)***S***_***0***_. We will deal with these initial values after infection later in the Section “The IFN- *γ*, upregulation and free virus model”. But now, we think of all the initial values as zero; i.e. ***I***_***0***_=0, ***U***_***0***_=0 and ***A***_***0***_=0. We still don’t expect to have any contribution to ***C*** ^′^(0), ***D*** ^′^(0) and ***N*** ^′^(0); i.e. *H*_1_(***I***_***0***_,***U***_***0***_,***A***_***0***_)=0, *H*_2_(***I***_***0***_,***U***_***0***_,***A***_***0***_)=0 and *H*_3_(***I***_***0***_,***U***_***0***_,***A***_***0***_)=0. We are interested in the deviation of ***C***, ***D*** and ***N*** from their optimal values ***C***_***0***_, ***D***_***0***_ and ***N***_***0***_ due to the changes ***i***, ***u*** and ***a*** from the base IFN- *γ*, upregulation and virus values. So let ***c***=***C***−***C***_***0***_, ***d***=***D***−***D***_***0***_ and ***n***=***N***−***N***_***0***_. We can then write ***C***=***C***_***0***_+***c***, ***D***=***D***_***0***_+***d*** and ***N***=***N***_***0***_+***n*** The model can then be rewritten as

$$\begin{array}{@{}rcl@{}} (\boldsymbol{C_{0}} + \boldsymbol{c})^{\prime}(t) &=& H_{1}(\boldsymbol{I_{0}} + \boldsymbol{i},\boldsymbol{U_{0}} + \boldsymbol{u}, \boldsymbol{A_{0}} + \boldsymbol{a}) \\ (\boldsymbol{D_{0}} + \boldsymbol{d})^{\prime}(t) &=& H_{2}(\boldsymbol{I_{0}} + \boldsymbol{i},\boldsymbol{U_{0}} + \boldsymbol{u}, \boldsymbol{A_{0}} + \boldsymbol{a})\\ (\boldsymbol{D_{0}} + \boldsymbol{d})^{\prime}(t) &=& H_{3}(\boldsymbol{I_{0}} + \boldsymbol{i},\boldsymbol{U_{0}} + \boldsymbol{u}, \boldsymbol{A_{0}} + \boldsymbol{a}) \end{array} $$

which, as usual, implies

$$\begin{array}{@{}rcl@{}} \boldsymbol{c}^{\prime}(t) &=& H_{1}(\boldsymbol{I_{0}} + \boldsymbol{i},\boldsymbol{U_{0}} + \boldsymbol{u}, \boldsymbol{A_{0}} + \boldsymbol{a}) \\ \boldsymbol{d}^{\prime}(t) &=& H_{2}(\boldsymbol{I_{0}} + \boldsymbol{i},\boldsymbol{U_{0}} + \boldsymbol{u}, \boldsymbol{A_{0}} + \boldsymbol{a})\\ \boldsymbol{d}^{\prime}(t) &=& H_{3}(\boldsymbol{I_{0}} + \boldsymbol{i},\boldsymbol{U_{0}} + \boldsymbol{u}, \boldsymbol{A_{0}} + \boldsymbol{a}) \end{array} $$

Next, we again perform a tangent plane approximation on the nonlinear dynamics functions *H*_1_, *H*_2_ and *H*_3_ just as we did in Section “Linearization details” for the *F*_1_, *F*_2_ and *F*_3_ we use for the ***CDN*** model.

### Linearization details

Once again, it seems reasonable to assume that since we are so close to ordinary operating conditions, the tangent plane errors are negligible. Letting ()^*o*^ denote that we are evaluating the partial derivatives at the point (***I***_***0***_,***U***_***0***_,***A***_***0***_), we find

$$\begin{array}{@{}rcl@{}} H_{1}(\boldsymbol{I_{0}} + \boldsymbol{i},\boldsymbol{U_{0}} + \boldsymbol{u}, \boldsymbol{A_{0}} + \boldsymbol{a}) &\approx& \left(\frac{\partial H_{1}}{\partial \boldsymbol{i}}\right)^{o} \boldsymbol{i}+ \left(\frac{\partial H_{1}}{\partial \boldsymbol{u}}\right)^{o} \boldsymbol{u}\\&&+ \left(\frac{\partial H_{1}}{\partial \boldsymbol{a}}\right)^{o} \boldsymbol{a}\\ H_{2}(\boldsymbol{I_{0}} + \boldsymbol{i},\boldsymbol{U_{0}} + \boldsymbol{u}, \boldsymbol{A_{0}} + \boldsymbol{a}) &\approx& \left(\frac{\partial H_{2}}{\partial \boldsymbol{i}}\right)^{o} \boldsymbol{i}+ \left(\frac{\partial H_{2}}{\partial \boldsymbol{u}}\right)^{o} \boldsymbol{u}\\&&+ \left(\frac{\partial H_{2}}{\partial \boldsymbol{a}}\right)^{o} \boldsymbol{a}\\ H_{3}(\boldsymbol{I_{0}} + \boldsymbol{i},\boldsymbol{U_{0}} + \boldsymbol{u}, \boldsymbol{A_{0}} + \boldsymbol{a}) &\approx& \left(\frac{\partial H_{3}}{\partial \boldsymbol{i}}\right)^{o} \boldsymbol{i}+ \left(\frac{\partial H_{3}}{\partial \boldsymbol{u}}\right)^{o} \boldsymbol{u}\\&&+ \left(\frac{\partial H_{3}}{\partial \boldsymbol{a}}\right)^{o} \boldsymbol{a}\\ \end{array} $$

The corresponding nonlinear dynamics approximation in matrix - vector form is then

$$\begin{array}{@{}rcl@{}} \left[\begin{array}{l} \boldsymbol{c}^{\prime}\\ \boldsymbol{d}^{\prime}\\ \boldsymbol{n}^{\prime} \end{array}\right] \approx \left[\begin{array}{lll} H_{1\boldsymbol{i}}^{o} & H_{1\boldsymbol{u}}^{o} & H_{1\boldsymbol{a}}^{o}\\ H_{2\boldsymbol{i}}^{o} & H_{2\boldsymbol{u}}^{o} & H_{2\boldsymbol{a}}^{o}\\ H_{3\boldsymbol{i}}^{o} & H_{3\boldsymbol{u}}^{o} & H_{3\boldsymbol{a}}^{o} \end{array}\right] \left[\begin{array}{l} \boldsymbol{i}\\ \boldsymbol{u}\\ \boldsymbol{a} \end{array}\right] \end{array} $$

where we now use a standard subscript scheme to indicate the partials. We therefore find the nonlinear dynamics approximation is

$$\begin{array}{@{}rcl@{}} \left[\begin{array}{l} \boldsymbol{c}^{\prime}\\ \boldsymbol{d}^{\prime}\\ \boldsymbol{n}^{\prime} \end{array}\right] \approx \left[\begin{array}{lll} H_{1\boldsymbol{i}}^{o} & H_{1\boldsymbol{u}}^{o} & H_{1\boldsymbol{a}}^{o}\\ H_{2\boldsymbol{i}}^{o} & H_{2\boldsymbol{u}}^{o} & H_{2\boldsymbol{a}}^{o}\\ H_{3\boldsymbol{i}}^{o} & H_{3\boldsymbol{u}}^{o} & H_{3\boldsymbol{a}}^{o} \end{array}\right] \left[\begin{array}{l} \boldsymbol{i}\\ \boldsymbol{u}\\ \boldsymbol{a} \end{array}\right] \end{array} $$

where we now use a standard subscript scheme to indicate the partials.

### The algebraic signs of the linearization matrix

If we hold everything constant except ***i*** which we increase to ***i***+*δ* ***i***, what happens? Increasing the IFN- *γ* level should increase collateral damage. Hence, $H_{1\boldsymbol {i}}^{o} = +$. What about the number of dividing cells that are infected? We do not think this should have an effect; hence, $H_{2\boldsymbol {i}}^{o} = 0$ too. A similar argument suggests $H_{3\boldsymbol {i}}^{o} = 0$ as well. Thus, the coefficient matrix above which we call ***Ψ*** so far looks like

$$\begin{array}{@{}rcl@{}} \boldsymbol{\Psi} &=&\left[\begin{array}{lll} + & H_{1\boldsymbol{u}}^{o} & H_{1\boldsymbol{a}}^{o} \\ 0 & H_{2\boldsymbol{u}}^{o} & H_{2\boldsymbol{a}}^{o} \\ 0 & H_{3\boldsymbol{u}}^{o} & H_{3\boldsymbol{a}}^{o} \end{array}\right] \end{array} $$

Now hold everything constant except the upregulation ***u*** and increase ***u*** to ***u***+*δ* ***u***. What happens? Increasing the upregulation level should increase collateral damage. Hence, $H_{1\boldsymbol {u}}^{o} = +$. Now infected cells are lysed once their IFN- *γ* upregulation hits a certain level and the increase in upregulation that makes the infected cells more visible certainly effects this as it will take fewer additional IFN- *γ* signals to trigger lysis. In general, there is a lag of say $T_{L}^{D}$ and $T_{L}^{N}$ time steps before this happens. With the increase *δ* ***u***, we expect $T_{L}^{D}$ and $T_{L}^{N}$ to both go down. Hence ***D*** and ***N*** decrease and we have $H_{2\boldsymbol {u}}^{o} = -$ and $H_{3\boldsymbol {u}}^{o} = -$ for each type of infected cell. Thus, the coefficient matrix looks like

$$\begin{array}{@{}rcl@{}} \boldsymbol{\Psi} &=&\left[\begin{array}{lll} + & + & H_{1\boldsymbol{a}}^{o} \\ 0 & - & H_{2\boldsymbol{a}}^{o} \\ 0 & - & H_{3\boldsymbol{a}}^{o} \end{array}\right] \end{array} $$

Next hold everything constant except the virus level ***a*** and increase ***a*** to ***a***+*δ* ***a***. What happens? Let’s think of the virus increase *δ* ***a*** as giving rise to an increase in the amount of virus stored inside a dividing cell or a non dividing cell. Now if the amount of virus in the cell goes up, that means when the cell is lysed, there is more virus available to infect cells which means more cells will be infected in later times. An increase in virus means an increase in collateral damage in general, so $H_{1\boldsymbol {a}}^{o} = +$. At a given time then, *A* is the virus level. We can write *A*=*A*_*F*_+*A*_*D*_+*A*_*N*_ where *A*_*F*_ is the free virus, *A*_*D*_ is the virus inside the dividing cells and *A*_*N*_ is the virus inside the nondividing cells. So if *A*_*D*_ goes up, we expect the amount of *A* and *A*_*F*_ to remain constant. Hence, if *A*_*D*_ goes up, *A*_*N*_ goes down.

The increase in *A*_*D*_ would mean more virus is released on lysis and hence an increase in ***D***. However, that increase in *A*_*D*_ is a concomitant decrease in ***N***. So we should have $H_{2\boldsymbol {a}}^{o} = +$ and $H_{3\boldsymbol {a}}^{o} = -$. Thus, the coefficient matrix now looks like

$$\begin{array}{@{}rcl@{}} \boldsymbol{\Psi} &=&\left[\begin{array}{lll} + & + & + \\ 0 & - & +\\ 0 & - & - \end{array}\right] \end{array} $$

Or letting $H_{2\boldsymbol {u}}^{o} = -a$, $H_{3\boldsymbol {u}}^{o} = -b$, $H_{2\boldsymbol {a}}^{o} = c$, $H_{3\boldsymbol {a}}^{o} = d$, the coefficient matrix now looks like

$$\begin{array}{@{}rcl@{}} \boldsymbol{\Psi} &=&\left[\begin{array}{lll} + & + & + \\ 0 & - a & c\\ 0 & - b & -d \end{array}\right] \end{array} $$

Thus, we have the changes in collateral damage and infection levels

$$\begin{array}{@{}rcl@{}} \boldsymbol{c}^{\prime} &=& H_{1\boldsymbol{i}}^{o} \: \boldsymbol{i} + H_{1\boldsymbol{u}}^{o} \: \boldsymbol{u} + H_{1\boldsymbol{a}}^{o} \: \boldsymbol{a}\\ \boldsymbol{d}^{\prime} &=& H_{2\boldsymbol{u}}^{o} \: \boldsymbol{u} + H_{2\boldsymbol{a}}^{o} \: \boldsymbol{a}\\ \boldsymbol{n}^{\prime} &=& H_{3\boldsymbol{u}}^{o} \: \boldsymbol{u} + H_{3\boldsymbol{a}}^{o} \: \boldsymbol{a} \end{array} $$

Now we need to estimate ***i***, ***u*** and ***a***.

## The IFN- *γ*, upregulation and free virus model

The amount of interferon level ***I***, the upregulation level ***U*** and the free virus level ***A*** depend on the initial amount of virus applied when in the equilibrium state; i.e. this is the amount that causes the initial infection. This is ***S***_***o***_. We assume the dynamics here are

$$\begin{array}{@{}rcl@{}} \boldsymbol{I}^{\prime} &=& G_{1}(\boldsymbol{I,U,A}) \\ \boldsymbol{U}^{\prime}&=& G_{2}(\boldsymbol{I,U,A}) \\ \boldsymbol{A}^{\prime}&=& G_{3}(\boldsymbol{I,U,A}) \end{array} $$

In the model of “The CDN IFN- *γ*, upregulation and virus model” section, we assumed ***C***, ***D*** and ***N*** depended on the perturbations of ***I***, ***U*** and ***A*** from a zero state. Now, we want to model the ***I***, ***U*** and ***A*** deviations from a base state ***I***_***0***_, ***U***_***0***_ and ***A***_***0***_ which is not zero. As previously discussed, we expect ***A***_***0***_=(1−*p*_0_) ***S***_***0***_, the initial IFN- *γ* level ***I***_***0***_=0 and the initial upregulation level ***U***_***0***_=*q*_1_ ***S***_***0***_. Let the deviations from these equilibrium values be given by ***i***=***I***−***I***_***0***_, ***u***=***U***−***U***_***0***_ and ***a***=***A***−***A***_***0***_. The model can then be rewritten as

$$\begin{array}{@{}rcl@{}} (\boldsymbol{I_{0}} + \boldsymbol{i})^{\prime}(t) &=& G_{1}(\boldsymbol{i}+\boldsymbol{I_{0}},\boldsymbol{u}+\boldsymbol{U_{0}}, \boldsymbol{a}+\boldsymbol{A_{0}}) \\ (\boldsymbol{U_{0}} + \boldsymbol{u})^{\prime}(t) &=& G_{2}(\boldsymbol{i}+\boldsymbol{I_{0}},\boldsymbol{u}+\boldsymbol{U_{0}}, \boldsymbol{a}+\boldsymbol{A_{0}}) \\ (\boldsymbol{A_{0}} + \boldsymbol{a})^{\prime}(t) &=& G_{3}(\boldsymbol{i}+\boldsymbol{I_{0}},\boldsymbol{u}+\boldsymbol{U_{0}}, \boldsymbol{a}+\boldsymbol{A_{0}}) \end{array} $$

or

$$\begin{array}{@{}rcl@{}} \boldsymbol{i}^{\prime}(t) &=& G_{1}(\boldsymbol{i}+\boldsymbol{I_{0}},\boldsymbol{u}+\boldsymbol{U_{0}}, \boldsymbol{a}+\boldsymbol{A_{0}}) \\ \boldsymbol{u}^{\prime}(t) &=& G_{2}(\boldsymbol{i}+\boldsymbol{I_{0}},\boldsymbol{u}+\boldsymbol{U_{0}}, \boldsymbol{a}+\boldsymbol{A_{0}}) \\ \boldsymbol{a}^{\prime}(t) &=& G_{3}(\boldsymbol{i}+\boldsymbol{I_{0}},\boldsymbol{u}+\boldsymbol{U_{0}}, \boldsymbol{a}+\boldsymbol{A_{0}}) \end{array} $$

The approximation to the ***i***, ***u*** and ***a*** model is handled in a way that is very similar to the previous two expansions which were explained in some detail in “Linearization details” section.

### Linearization details

The linearization again involves a tangent plane approximation to nonlinear dynamics functions, which are now the functions *G*_1_, *G*_2_ and *G*_3_. It seems reasonable to assume that since we are so close to ordinary operating conditions, the tangent plane errors as usual will be negligible. Thus, using $()^{\boldsymbol {S_{0}}}\phantom {\dot {i}\!}$ to denote the partials evaluated at the base point the model approximation, we have

$$\begin{array}{@{}rcl@{}} G_{1}(\boldsymbol{i},\boldsymbol{u}, \boldsymbol{a}) &\approx& \left(\frac{\partial G_{1}}{\partial \boldsymbol{i}}\right)^{\boldsymbol{S_{0}}} \boldsymbol{i}+ \left(\frac{\partial G_{1}}{\partial \boldsymbol{u}}\right)^{\boldsymbol{S_{0}}} \boldsymbol{u}+ \left(\frac{\partial G_{1}}{\partial \boldsymbol{a}}\right)^{\boldsymbol{S_{0}}} \boldsymbol{a}\\ G_{2}(\boldsymbol{i},\boldsymbol{u}, \boldsymbol{a}) &\approx& \left(\frac{\partial G_{2}}{\partial \boldsymbol{i}}\right)^{\boldsymbol{S_{0}}} \boldsymbol{i}+ \left(\frac{\partial G_{2}}{\partial \boldsymbol{u}}\right)^{\boldsymbol{S_{0}}} \boldsymbol{u}+ \left(\frac{\partial G_{2}}{\partial \boldsymbol{a}}\right)^{\boldsymbol{S_{0}}} \boldsymbol{a}\\ G_{3}(\boldsymbol{i},\boldsymbol{u}, \boldsymbol{a}) &\approx& \left(\frac{\partial G_{3}}{\partial \boldsymbol{i}}\right)^{\boldsymbol{S_{0}}} \boldsymbol{i}+ \left(\frac{\partial G_{3}}{\partial \boldsymbol{u}}\right)^{\boldsymbol{S_{0}}} \boldsymbol{u}+ \left(\frac{\partial G_{3}}{\partial \boldsymbol{a}}\right)^{\boldsymbol{S_{0}}} \boldsymbol{a} \end{array} $$

Thus our dynamics approximation in matrix - vector form is

$$\begin{array}{@{}rcl@{}} \left[\begin{array}{l} \boldsymbol{i}^{\prime}\\ \boldsymbol{u}^{\prime}\\ \boldsymbol{d}^{\prime} \end{array}\right] \approx \left[\begin{array}{lll} G_{1\boldsymbol{i}}^{\boldsymbol{S_{0}}} & G_{1\boldsymbol{u}}^{\boldsymbol{S_{0}}} & G_{1\boldsymbol{a}}^{\boldsymbol{S_{0}}} \\ G_{2\boldsymbol{i}}^{\boldsymbol{S_{0}}} & G_{2\boldsymbol{u}}^{\boldsymbol{S_{0}}} & G_{2\boldsymbol{a}}^{\boldsymbol{S_{0}}} \\ G_{3\boldsymbol{i}}^{\boldsymbol{S_{0}}} & G_{3\boldsymbol{u}}^{\boldsymbol{S_{0}}} & G_{3\boldsymbol{a}}^{\boldsymbol{S_{0}}} \end{array}\right] \left[\begin{array}{l} \boldsymbol{i}\\ \boldsymbol{u}\\ \boldsymbol{a} \end{array}\right] \end{array} $$

The dynamics approximation is then given by

$$\begin{array}{@{}rcl@{}} \left[\begin{array}{l} \boldsymbol{i}^{\prime}\\ \boldsymbol{u}^{\prime}\\ \boldsymbol{d}^{\prime} \end{array}\right] \approx \left[\begin{array}{lll} G_{1\boldsymbol{i}}^{\boldsymbol{S_{0}}} & G_{1\boldsymbol{u}}^{\boldsymbol{S_{0}}} & G_{1\boldsymbol{a}}^{\boldsymbol{S_{0}}} \\ G_{2\boldsymbol{i}}^{\boldsymbol{S_{0}}} & G_{2\boldsymbol{u}}^{\boldsymbol{S_{0}}} & G_{2\boldsymbol{a}}^{\boldsymbol{S_{0}}} \\ G_{3\boldsymbol{i}}^{\boldsymbol{S_{0}}} & G_{3\boldsymbol{u}}^{\boldsymbol{S_{0}}} & G_{3\boldsymbol{a}}^{\boldsymbol{S_{0}}} \end{array}\right] \left[\begin{array}{l} \boldsymbol{i}\\ \boldsymbol{u}\\ \boldsymbol{a} \end{array}\right] \end{array} $$

### The algebraic signs of the linearization matrix

The analysis of the signs of these partials is next. This is similar to what we did for the previous model. If we hold everything constant except ***i*** which we increase to ***i***+*δ* ***i***, what happens? Increasing the IFN- *γ* level should increase IFN- *γ*. Hence, $G_{1\boldsymbol {i}}^{\boldsymbol {S_{0}}} = +$. What about the upregulation level? We do not think this should have an effect; hence, $G_{2\boldsymbol {i}}^{\boldsymbol {S_{0}}} = 0$ too. A similar argument suggests $G_{3\boldsymbol {i}}^{\boldsymbol {S_{0}}} = 0$ as well. Thus, the coefficient matrix above which we call ***Φ*** so far looks like

$$\begin{array}{@{}rcl@{}} \boldsymbol{\Phi} &=&\left[\begin{array}{lll} + & G_{1\boldsymbol{u}}^{\boldsymbol{S_{0}}} & G_{1\boldsymbol{a}}^{\boldsymbol{S_{0}}} \\ 0 & G_{2\boldsymbol{u}}^{\boldsymbol{S_{0}}} & G_{2\boldsymbol{a}}^{\boldsymbol{S_{0}}} \\ 0 & G_{3\boldsymbol{u}}^{\boldsymbol{S_{0}}} & G_{3\boldsymbol{a}}^{\boldsymbol{S_{0}}} \end{array}\right] \end{array} $$

Now hold everything constant except the upregulation ***u*** and increase ***u*** to ***u***+*δ* ***u***. What happens? Increasing the upregulation level should increase the IFN- *γ* level. Hence, $G_{1\boldsymbol {u}}^{\boldsymbol {S_{0}}} = +$. If upregulation increases, the ***u*** ^′^ goes up, hence $G_{2\boldsymbol {u}}^{\boldsymbol {S_{0}}} = +$. What about the level of virus? If upregulation increases, the increase in upregulation makes infected cells more visible as it will take fewer additional IFN- *γ* signals to trigger lysis. In general, there is a lag of say $T_{L}^{D}$ and $T_{L}^{N}$ time steps before this happens. With the increase *δ* ***u***, we expect $T_{L}^{D}$ and $T_{L}^{N}$ to both go down. Hence, free virus should increase; i.e. $G_{3\boldsymbol {u}}^{\boldsymbol {S_{0}}} = +$. Thus, the coefficient matrix looks like

$$\begin{array}{@{}rcl@{}} \boldsymbol{\Phi} &=&\left[\begin{array}{lll} + & + & G_{1\boldsymbol{a}}^{\boldsymbol{S_{0}}} \\ 0 & + & G_{2\boldsymbol{a}}^{\boldsymbol{S_{0}}} \\ 0 & + & G_{3\boldsymbol{a}}^{\boldsymbol{S_{0}}} \end{array}\right] \end{array} $$

Now hold everything constant except the virus level ***a*** and increase ***a*** to ***a***+*δ* ***a***. What happens? Let’s think of the virus increase *δ* ***a*** as giving rise to an increase in the amount of virus stored inside a dividing cell or a non dividing cell. Now if the amount of virus in the cell goes up, that means when the cell is lysed, there is more virus available to infect cells which means more cells will be infected in later times. An increase in virus should not effect IFN- *γ* levels, so $G_{1\boldsymbol {a}}^{\boldsymbol {S_{0}}} = 0$. An increase in virus must imply $G_{3\boldsymbol {a}}^{\boldsymbol {S_{0}}} = +$ Now reason as we did earlier: at a given time then, *A* is the free virus level. We can write *A*=*A*_*F*_+*A*_*D*_+*A*_*N*_ where *A*_*F*_ is the free virus, *A*_*D*_ is the virus inside the dividing cells and *A*_*N*_ is the virus inside the nondividing cells. So if *A*_*F*_ goes up and the total amount of virus at this time is constant, we expect *A*_*D*_ and *A*_*N*_ to go down. If the amount of virus inside the cells decreases, we expect the upregulation to decrease. So $G_{2\boldsymbol {a}}^{\boldsymbol {S_{0}}} = -$. Thus, the coefficient matrix now looks like

$$\begin{array}{@{}rcl@{}} \boldsymbol{\Phi} &=&\left[\begin{array}{lll} + & + & 0 \\ 0 & + & -\\ 0 & + & + \end{array}\right] \end{array} $$

Or letting $G_{2\boldsymbol {u}}^{\boldsymbol {S_{0}}} = a$, $G_{3\boldsymbol {u}}^{\boldsymbol {S_{0}}} = b$, $G_{2\boldsymbol {a}}^{\boldsymbol {S_{0}}} = -c$, $G_{3\boldsymbol {a}}^{\boldsymbol {S_{0}}} = d$, we have the coefficient matrix now looks like

$$\begin{array}{@{}rcl@{}} \boldsymbol{\Phi} &=&\left[\begin{array}{lll} + & + & 0 \\ 0 & a & -c\\ 0 & b & d \end{array}\right] \end{array} $$

## Oscillations in upregulation and free virus

We now use standard results from the theory of linear differential equation systems. This analysis relies on the eigenvalues of our model systems. The eigenvalues of this linearized system are found by solving the det(*λ* ***I***−***Φ***)=0. Thus, for the coefficient matrix *Φ*, we have

$$\begin{array}{@{}rcl@{}} \det(\lambda \boldsymbol{I} - \boldsymbol{\Phi}) &=&\det \: \left[\begin{array}{ccc} \lambda - G_{1\boldsymbol{i}}^{\boldsymbol{S_{0}}}& -G_{1\boldsymbol{u}}^{\boldsymbol{S_{0}}} & -G_{1\boldsymbol{a}}^{\boldsymbol{S_{0}}} \\ 0 & \lambda - G_{2\boldsymbol{u}}^{\boldsymbol{S_{0}}} & -G_{2\boldsymbol{a}}^{\boldsymbol{S_{0}}} \\ 0 & -G_{3\boldsymbol{u}}^{\boldsymbol{S_{0}}} & \lambda - G_{3\boldsymbol{a}}^{\boldsymbol{S_{0}}} \end{array}\right] = 0. \end{array} $$

This gives

$$\begin{array}{@{}rcl@{}} & & 0 = \left(\lambda - G_{1\boldsymbol{i}}^{\boldsymbol{S_{0}}} \right) \: \left(\left(\lambda - G_{2\boldsymbol{u}}^{\boldsymbol{S_{0}}}\right) \left(\lambda - G_{3\boldsymbol{a}}^{\boldsymbol{S_{0}}}\right) - G_{2\boldsymbol{u}}^{\boldsymbol{S_{0}}} G_{3\boldsymbol{a}}^{\boldsymbol{S_{0}}} \right) \end{array} $$

The eigenvalues of the two by two submatrix are the most interesting. Consider the determinant

$$\begin{array}{@{}rcl@{}} \det\left[\begin{array}{cc} \lambda - \alpha & -\beta \\ \beta & \lambda - \alpha \end{array}\right] = \lambda^{2} - 2 \alpha \lambda + \alpha^{2} + \beta^{2} \end{array} $$

This has the complex roots (here the symbol ***j*** is defined to be $j = \sqrt {-1}$).

$$\begin{array}{@{}rcl@{}} \lambda_{1} &=& \alpha + \beta \boldsymbol{j}, \quad \lambda_{2} = \alpha - \beta \boldsymbol{j} \end{array} $$

Hence, we can get complex roots if

$$\begin{array}{@{}rcl@{}} \left[\begin{array}{ll} G_{2\boldsymbol{u}}^{\boldsymbol{S_{0}}} & G_{2\boldsymbol{a}}^{\boldsymbol{S_{0}}}\\ G_{3\boldsymbol{u}}^{\boldsymbol{S_{0}}} & G_{3\boldsymbol{a}}^{\boldsymbol{S_{0}}} \end{array}\right] = \left[\begin{array}{ll} \alpha & - \beta\\ \beta & \alpha \end{array}\right] \end{array} $$

or $G_{2\boldsymbol {u}}^{\boldsymbol {S_{0}}} = G_{3\boldsymbol {a}}^{\boldsymbol {S_{0}}}$ and $G_{3\boldsymbol {u}}^{\boldsymbol {S_{0}}} = -G_{2\boldsymbol {a}}^{\boldsymbol {S_{0}}}$. Since an eigenvalue equation of the form

$$\begin{array}{@{}rcl@{}} \det \left[\begin{array}{ll} \lambda -a & c \\ -b & \lambda - d \end{array}\right] = \lambda^{2} - (a + d) \lambda + ad + bc \end{array} $$

has roots

$$\begin{array}{@{}rcl@{}} \lambda &=& \frac{(d + a) \pm \sqrt{(d+a)^{2} -4(ad+bc)}}{2}\\ &=& \frac{(d + a) \pm \sqrt{d^{2} - 2ad + a^{2} - 4bc}}{2}, \end{array} $$

we have complex roots if (*a*−*d*)^2^−4*b* *c*<0. In particular, if *a*=*d*=*α* and *b*=*β* and *c*=*β*, then we have −4*β* ^2^<0 and the roots are complex. We can have complex roots for other choices of *a*, *b*, *c* and ***D*** which implies conditions on the partials which is something we can explore, but we will not do that here. For our purposes, we will examine closely what happens when $a = G_{2\boldsymbol {u}}^{\boldsymbol {S_{0}}} = d = G_{3\boldsymbol {a}}^{\boldsymbol {S_{0}}}$ and $b = G_{3\boldsymbol {u}}^{\boldsymbol {S_{0}}} = -c = -G_{2\boldsymbol {a}}^{\boldsymbol {S_{0}}}$ The general solution to the system with the complex eigenvalues we have been discussing is this. The eigenvalues are $\lambda _{1} = G_{1\boldsymbol {i}}^{o}$ and the complex conjugate pair *α*±*β* ***j*** where $\alpha = G_{2\boldsymbol {u}}^{\boldsymbol {S_{0}}} = G_{3\boldsymbol {a}}^{\boldsymbol {S_{0}}}$ and $\beta = G_{3\boldsymbol {u}}^{\boldsymbol {S_{0}}} = -G_{2\boldsymbol {a}}^{\boldsymbol {S_{0}}}$. The eigenvectors here are simple

$$\begin{array}{@{}rcl@{}} \boldsymbol{V} + \boldsymbol{j} \boldsymbol{W} &=& \left[\begin{array}{l} 1\\ 0 \end{array}\right] + \boldsymbol{j} \left[\begin{array}{l} 0 \\ 1 \end{array}\right], \quad \quad \boldsymbol{V} - \boldsymbol{j} \boldsymbol{W} = \left[\begin{array}{l} 1\\ 0 \end{array}\right] - \boldsymbol{j} \left[\begin{array}{l} 0 \\ 1 \end{array}\right] \end{array} $$

We can then solve for ***u*** and ***a*** to find

$$\begin{array}{@{}rcl@{}} & & \left[\begin{array}{l} \boldsymbol{u}(t)\\ \boldsymbol{a}(t) \end{array}\right] = e^{\alpha t} \left[\begin{array}{l} (a \cos(\beta t) + b \sin(\beta t)\\ (b \cos(\beta t) -a \sin(\beta t) \end{array}\right] \end{array} $$

Letting $R = \sqrt {a^{2}+b^{2}}$, we find

$$\begin{array}{@{}rcl@{}} & & \left[\begin{array}{l} u(t)\\ a(t) \end{array}\right] = R e^{\alpha t} \left[\begin{array}{l} \cos(\beta t - \delta)\\ -\sin(\beta t - \delta) \end{array}\right] \end{array} $$

where *δ* is defined as tan−1(*b*/*a*). The full solution is then

$$\begin{array}{@{}rcl@{}} & & \left[\begin{array}{l} \boldsymbol{i}(t) \\ \boldsymbol{u}(t)\\ \boldsymbol{a}(t) \end{array}\right] = \left[\begin{array}{l} A e^{-G_{1\boldsymbol{i}}^{o} t}\\ R e^{\alpha t} \cos(\beta t - \delta)\\ -R e^{-\alpha t} \sin(\beta t - \delta) \end{array}\right] \end{array} $$

where *A*, *R*, $G_{1\boldsymbol {i}}^{o}$, *β* and *δ* determine a given model.

Here, we have ***u***_***0***_=*q*_1_ ***S***_***0***_ and ***a***_***0***_=(1−*p*_0_)***S***_***0***_. Hence, we roughly know at the time of the infection

$$\begin{array}{@{}rcl@{}} q_{1} \boldsymbol{S_{0}} &=& \left. \left (R e^{\alpha t} \cos(\beta t - \delta \right) \right|_{t=0} \: = \: R \cos(\delta)\\ (1-p_{0}) \boldsymbol{S_{0}} &=& -\left. \left (R e^{-\alpha t} \sin(\beta t - \delta) \right) \right|_{t=0} \: =\: R \sin(\delta) \end{array} $$

Taking a ratio, we find

$$\begin{array}{@{}rcl@{}} \tan(\delta) &=& \frac{\boldsymbol{a_{0}}}{\boldsymbol{u_{0}}} \: = \: \frac{(1-p_{0}) \boldsymbol{S_{0}}}{q_{1} \boldsymbol{S_{0}}} \: = \: \frac{(1-p_{0})}{q_{1}}. \end{array} $$

Hence, $\delta = \tan ^{-1}\left (\frac {(1-p_{0})}{q_{1}} \right)$ and

$$\begin{array}{@{}rcl@{}} R = \boldsymbol{u_{0}} \sec(\delta) = \sqrt{\boldsymbol{a_{0}}^{2} + \boldsymbol{u_{0}}^{2}} \: = \: \boldsymbol{S_{0}} \: \sqrt{q_{1}^{2} + (1-p_{0})^{2}}. \end{array} $$

Finally, recall we have $\alpha = G_{2\boldsymbol {u}}^{\boldsymbol {S_{0}}}$ and $\beta = G_{3\boldsymbol {u}}^{\boldsymbol {S_{0}}}$; thus, the oscillatory solutions for upregulation and free virus are

$$\begin{array}{@{}rcl@{}} & & \left[\begin{array}{l} \boldsymbol{u}(t)\\ \boldsymbol{a}(t) \end{array}\right] = \boldsymbol{S_{0}} \: \sqrt{q_{1}^{2} + (1-p_{0})^{2}} \: e^{G_{2\boldsymbol{u}}^{\boldsymbol{S_{0}}} t}\\ &&\left[\begin{array}{l} \cos \left (G_{3\boldsymbol{u}}^{\boldsymbol{S_{0}}} t - \tan^{-1}\left(\frac{(1-p_{0})}{q_{1}} \right) \right)\\ -\sin \left (G_{3\boldsymbol{u}}^{\boldsymbol{S_{0}}} t - \tan^{-1}\left(\frac{(1-p_{0})}{q_{1}} \right) \right) \end{array}\right] \end{array} $$

It seems unreasonable to us that the phase shift *δ* should be a constant; i.e. independent of ***S***_***0***_. After all, the reasoning above is approximate and we should not think of this as actually fixed. So we will assume that all critical parameters here are proportional to ***S***_***0***_. Our rough calculation showed us $R = \boldsymbol {S_{0}} \sqrt {q_{1}^{2} + (1-p_{0})^{2}}$, so it seems reasonable that *R* is proportional to ***S***_***0***_ in general. Therefore, we now assume

$$\begin{array}{@{}rcl@{}} R^{\boldsymbol{S_{0}}} &\propto& \boldsymbol{S_{0}} \: \Longrightarrow \: R^{\boldsymbol{S_{0}}} \: = \: r_{1} \: \boldsymbol{S_{0}}\\ G_{2\boldsymbol{u}}^{\boldsymbol{S_{0}}} &\propto& \boldsymbol{S_{0}} \: \Longrightarrow \: G_{2\boldsymbol{u}}^{\boldsymbol{S_{0}}} \: = \: r_{2} \: \boldsymbol{S_{0}}\\ G_{3\boldsymbol{u}}^{\boldsymbol{S_{0}}} &\propto& \boldsymbol{S_{0}} \: \Longrightarrow \: G_{3\boldsymbol{u}}^{\boldsymbol{S_{0}}} \: = \: r_{3} \: \boldsymbol{S_{0}}\\ \delta^{\boldsymbol{S_{0}}} &\propto& \boldsymbol{S_{0}} \: \Longrightarrow \: \delta^{\boldsymbol{S_{0}}} \: = \: r_{4} \: \boldsymbol{S_{0}} \end{array} $$

for a new parameters *r*_1_, *r*_2_, *r*_3_ and *r*_4_. This leads to our estimate of the dependence of the upregulation and free virus on the initial dose ***S***_***0***_: we have

$$\begin{array}{@{}rcl@{}} & & \left[\begin{array}{l} u(t)\\ a(t)\end{array}\right] = r_{1} \boldsymbol{S_{0}} \: \: e^{r_{2} \boldsymbol{S_{0}} \: t} \left[\begin{array}{l} \cos (r_{3} \boldsymbol{S_{0}} \: t - r_{4} \boldsymbol{S_{0}}) \\ -\sin (r_{3} \boldsymbol{S_{0}} \: t - r_{4} \boldsymbol{S_{0}}) \end{array}\right] \end{array} $$

## A health model

Roughly speaking, if the total number of cells is ***T***, the number of healthy cells can be approximated by

$$\begin{array}{@{}rcl@{}} \boldsymbol{H} &=& \boldsymbol{T} - (C_{0} + c(t)) - (D_{0} + d(t)) - (N_{0} + n(t)) \end{array} $$

We know

$$\begin{array}{@{}rcl@{}} \boldsymbol{c}^{\prime} &=& H_{1\boldsymbol{i}}^{o} \: \boldsymbol{i} + H_{1\boldsymbol{u}}^{o} \: \boldsymbol{u} + H_{1\boldsymbol{a}}^{o} \: \boldsymbol{a}\\ \boldsymbol{d}^{\prime} &=& H_{2\boldsymbol{u}}^{o} \: \boldsymbol{u} + H_{2\boldsymbol{a}}^{o} \: \boldsymbol{a}\\ \boldsymbol{n}^{\prime} &=& H_{3\boldsymbol{u}}^{o} \: \boldsymbol{u} + H_{3\boldsymbol{a}}^{o} \: \boldsymbol{a} \end{array} $$

and so we are looking at deviations from the base values ***I***_***0***_=0, ***U***_***0***_=*q*_1_ ***S***_***0***_ and ***A***_***0***_=(1−*p*_0_)***S***_***0***_. It follows we have

$$\begin{array}{@{}rcl@{}} \boldsymbol{C}(t) &=& \boldsymbol{C_{0}} + H_{1\boldsymbol{i}}^{o} \: \left (\int_{0}^{t} \boldsymbol{i}(s) ds \right)\\ &&+ H_{1\boldsymbol{u}}^{o} \: \left (q_{1} \boldsymbol{S_{0}} + \int_{0}^{t} \boldsymbol{u}(s) ds \right)\\ &&+ H_{1\boldsymbol{a}}^{o} \: \left ((1-p_{0}) \boldsymbol{S_{0}} + \int_{0}^{t} \boldsymbol{a}(s) ds \right)\\ \boldsymbol{D}(t) &=& \boldsymbol{D_{0}} + H_{2\boldsymbol{u}}^{o} \: \left(q_{1} \boldsymbol{S_{0}} + \int_{0}^{t} \boldsymbol{u}(s) ds \right)\\ &&+ H_{2\boldsymbol{a}}^{o} \: \left((1-p_{0}) \boldsymbol{S_{0}} + \int_{0}^{t} \boldsymbol{a}(s) ds \right)\\ \boldsymbol{N}(t) &=& \boldsymbol{N_{0}} + H_{3\boldsymbol{u}}^{o} \: \left(q_{1} \boldsymbol{S_{0}} + \int_{0}^{t} \boldsymbol{u}(s) ds \right)\\ &&+ H_{3\boldsymbol{a}}^{o} \: \left((1-p_{0}) \boldsymbol{S_{0}} + \int_{0}^{t} \boldsymbol{a}(s) ds \right) \end{array} $$

As discussed earlier, we have initially, ***C***_***0***_=0, ***D***_***0***_=*p*_2_ *p*_0_ ***S***_***0***_ and ***N***_***0***_=*p*_2_ *p*_0_ ***S***_***0***_. So we have

$$\begin{array}{@{}rcl@{}} \boldsymbol{C}(t) &=& H_{1\boldsymbol{i}}^{o} \: \left (\int_{0}^{t} \boldsymbol{i}(s) ds \right) + H_{1\boldsymbol{u}}^{o} \: \left (q_{1} \boldsymbol{S_{0}} + \int_{0}^{t} \boldsymbol{u}(s) ds \right)\\ &\quad +& H_{1\boldsymbol{a}}^{o} \: \left ((1-p_{0}) \boldsymbol{S_{0}} + \int_{0}^{t} \boldsymbol{a}(s) ds \right)\\ \boldsymbol{D}(t) &=& p_{2} \: p_{0} \: \boldsymbol{S_{0}} + H_{2\boldsymbol{u}}^{o} \: \left(q_{1} \boldsymbol{S_{0}} + \int_{0}^{t} \boldsymbol{u}(s) ds \right)\\ &&+ H_{2\boldsymbol{a}}^{o} \: \left((1-p_{0}) \boldsymbol{S_{0}} + \int_{0}^{t} \boldsymbol{a}(s) ds \right)\\ \boldsymbol{N}(t) &=& p_{2} \: p_{0} \: \boldsymbol{S_{0}} + H_{3\boldsymbol{u}}^{o} \: \left(q_{1} \boldsymbol{S_{0}} + \int_{0}^{t} \boldsymbol{u}(s) ds \right)\\ &&+ H_{3\boldsymbol{a}}^{o} \: \left((1-p_{0}) \boldsymbol{S_{0}} + \int_{0}^{t} \boldsymbol{a}(s) ds \right) \end{array} $$

Thus, we have

$${} \begin{aligned} \boldsymbol{H}(t) \!&=\! \boldsymbol{T} \,-\,\left(p_{1} \: p_{0} \: \boldsymbol{S_{0}} + p_{2} \: p_{0} \: \boldsymbol{S_{0}}\right) \,-\,\left(H_{1\boldsymbol{u}}^{o} \,+\,H_{2\boldsymbol{u}}^{o} + H_{3\boldsymbol{u}}^{o} \right) q_{1} \boldsymbol{S_{0}}\\ &\quad-\left(H_{1\boldsymbol{a}}^{o} + H_{2\boldsymbol{a}}^{o} + H_{3\boldsymbol{a}}^{o} \right) \: (1-p_{0}) \boldsymbol{S_{0}} - H_{1\boldsymbol{i}}^{o} \: \int_{0}^{t} \boldsymbol{i}(s) ds\\ &\quad-\!\left(\!H_{1\boldsymbol{u}}^{o} + H_{2\boldsymbol{u}}^{o} + H_{3\boldsymbol{u}}^{o} \right) \int_{0}^{t} \!\boldsymbol{u}(s) ds\! -\!\left(H_{1\boldsymbol{a}}^{o} \,+\, H_{2\boldsymbol{a}}^{o} \,+\, H_{3\boldsymbol{a}}^{o} \right)\\ &\quad\times \left(\int_{0}^{t} \boldsymbol{a}(s) ds \right) \end{aligned} $$

Now collect all the terms involving ***S***_***0***_ and set that coefficient to *Λ* for convenience. Making this replacement, we have

$$\begin{array}{@{}rcl@{}} \Lambda &=& (p_{1} + p_{2}) p_{0} + \left(H_{1\boldsymbol{u}}^{o} + H_{2\boldsymbol{u}}^{o} + H_{3\boldsymbol{u}}^{o} \right) \: q_{1}\\ &&+\left(H_{1\boldsymbol{a}}^{o} + H_{2\boldsymbol{a}}^{o} + H_{3\boldsymbol{a}}^{o} \right) \: (1-p_{0}) \end{array} $$

This leads to the simplification

$$\begin{array}{@{}rcl@{}} \boldsymbol{H}(t) &=& \!\!\boldsymbol{T} - \Lambda \boldsymbol{S_{0}} - H_{1\boldsymbol{i}}^{o} \int_{0}^{t} \boldsymbol{i}(s) ds -\left(H_{1\boldsymbol{u}}^{o} + H_{2\boldsymbol{u}}^{o} + H_{3\boldsymbol{u}}^{o} \right)\\ &&\int_{0}^{t} \boldsymbol{u}(s) ds\\ &\quad-&\left(H_{1\boldsymbol{a}}^{o} + H_{2\boldsymbol{a}}^{o} + H_{3\boldsymbol{a}}^{o} \right) \: \left(\int_{0}^{t} \boldsymbol{a}(s) ds \right) \end{array} $$

Now we have to compute these integrated transient values. We label them as ***IT*** for the transient ***i*** integration; ***UT*** for the transient ***u*** integration; and ***AT*** for the transient ***a*** integration. We then have

$$\begin{array}{@{}rcl@{}} \boldsymbol{IT}(t) &=& \int_{0}^{t} \boldsymbol{i}(s) ds = \int_{0}^{t} \: A e^{-G_{1\boldsymbol{i}}^{o} s} \: ds\\ \boldsymbol{UT}(t) &=& \int_{0}^{t} \boldsymbol{u}(s) ds = \int_{0}^{t} \: R e^{\alpha t} \cos(\beta s - \delta) \: ds\\ \boldsymbol{AT}(t) &=& \int_{0}^{t} \boldsymbol{a}(s) ds = - \int_{0}^{t} \: R e^{-\alpha t} \sin(\beta s - \delta) \end{array} $$

The ***i*** integration is straightforward

$$\begin{array}{@{}rcl@{}} \boldsymbol{IT}(t) &=& \int_{0}^{t} \boldsymbol{i}(s) ds = \int_{0}^{t} \: A e^{-G_{1\boldsymbol{i}}^{\boldsymbol{S_{0}}} s} \: ds =\frac{A}{G_{1\boldsymbol{i}}^{\boldsymbol{S_{0}}}} \left(1 - e^{-G_{1\boldsymbol{i}}^{\boldsymbol{S_{0}}} t} \right) \end{array} $$

however, the ***UT*** integration are more complicated.

### The ***UT*** calculation

To evaluate this term, we use integration by parts. Now we have to compute the integrated transient values required to find the health estimate. We have labeled the integrations as ***UT*** for the transient ***u*** integration; and ***AT*** for the transient ***a*** integration. We then have

$$\begin{array}{@{}rcl@{}} \boldsymbol{UT}(t) &=& \int_{0}^{t} \boldsymbol{u}(s) ds = \int_{0}^{t} \: R e^{\alpha t} \cos(\beta s - \delta) \: ds\\ \boldsymbol{AT}(t) &=& \int_{0}^{t} \boldsymbol{a}(s) ds = - \int_{0}^{t} \: R e^{-\alpha t} \sin(\beta s - \delta) \end{array} $$

### Integration details

First, let’s calculate ***UT***. To evaluate this term, we use integration by parts. We find

$$\begin{array}{@{}rcl@{}} \int_{0}^{t} \: e^{as} \cos(bs - c) &=& \left. \left(\frac{1}{b} \: e^{as} \: \sin(bs-c) \right) \right |_{0}^{t}\\ &&- \frac{a}{b} \int_{0}^{t} \: e^{as} \: \sin(bs -c) \: ds\\ &=& \frac{1}{(a^{2} + b^{2})} e^{at} (b \sin(bt-c) \\ &&+ a \cos(bt-c)) \\ &&+ \frac{1}{a^{2}+b^{2}} \left(b \sin(c) - a \cos(c) \right) \end{array} $$

and so we can find ***UT*** as follows:

$$\begin{array}{@{}rcl@{}} \boldsymbol{UT}(t) &=& \int_{0}^{t} \boldsymbol{u}(s) ds = \int_{0}^{t} \: R e^{\alpha t} \cos(\beta s - \delta) \: ds\\ &=& \frac{R}{\left(\alpha^{2} + \beta^{2}\right)} \: (e^{\alpha t} \left(\beta \sin(\beta t - \delta) + \alpha \cos(\beta t -\delta)\right)\\ [6pt] && + \left(\beta \sin(\delta) - \alpha \cos(\delta) \right)) \end{array} $$

We can rewrite this is a much better form using our assumptions. First, rewrite as

$${} {\begin{aligned} \boldsymbol{UT}(t) &= \frac{R}{\sqrt{\alpha^{2} + \beta^{2}}} e^{\alpha t} \left(\frac{\beta}{\sqrt{\alpha^{2} + \beta^{2}}} \left({\vphantom{\frac{\alpha}{\sqrt{\alpha^{2} + \beta^{2}}} \cos(\beta t -\delta)}}\sin(\beta t - \delta)\right.\right.\\ &\left.\quad+ \frac{\alpha}{\sqrt{\alpha^{2} + \beta^{2}}} \cos(\beta t -\delta)\right)\\ &\quad + \frac{R}{\sqrt{\alpha^{2}+\!\beta^{2}}} \!\left(\frac{\beta}{\sqrt{\alpha^{2} +\! \beta^{2}}} \sin(\delta) - \frac{\alpha}{\sqrt{\alpha^{2} + \beta^{2}}} \cos(\delta) \right) \end{aligned}} $$

Now we find ***AT***. Another standard integration by parts gives

$$\begin{array}{@{}rcl@{}} \int_{0}^{t} \: e^{as} \sin(bs - c) &=& \left. \left(- \frac{1}{b} \: e^{as} \: \cos(bs-c) \right) \right |_{0}^{t}\\ &&+ \frac{a}{b} \int_{0}^{t} \: e^{as} \: \cos(bs -c) \: ds\\ &=& \frac{1}{\left(a^{2} + b^{2}\right)} \: e^{at} (-b \cos(bt-c)\\ &&+ a \sin(bt-c))\\ &&+ \frac{1}{a^{2} + b^{2}} \left(b \cos(c) + a \sin(c) \right) \end{array} $$

and so we can find ***AT*** also:

$$\begin{array}{@{}rcl@{}} \boldsymbol{AT}(t) &=& \int_{0}^{t} \boldsymbol{a}(s) ds = - \int_{0}^{t} \: R e^{-\alpha t} \sin(\beta s - \delta)\\ &=& \!-\frac{R}{\left(\alpha^{2} + \beta^{2}\right)} e^{\alpha t}\! \left(-\beta \cos(\beta t - \delta) + \alpha \sin(\beta t -\delta) \right)\\ && - \frac{R}{\alpha^{2}+\beta^{2}} \left(\beta \cos(\delta) + \alpha \sin(\delta) \right) \end{array} $$

### Model results

We know *α*, *β*, *δ* and *R* are really dependent of ***S***_***0***_. For convenience of exposition, we drop the superscript ***S***_***0***_ in our calculations below

$$\begin{array}{@{}rcl@{}} \frac{R}{\sqrt{\alpha^{2} + \beta^{2}}} &=& \frac{r_{1} \boldsymbol{S_{0}}}{\sqrt{r_{2}^{2} + r_{3}^{2}} \boldsymbol{S_{0}}} = \frac{r_{1}}{\sqrt{r_{2}^{2}+r_{3}^{2}}}, \: \frac{\alpha}{\sqrt{\alpha^{2} + \beta^{2}}}\\ &=& \frac{r_{2} \boldsymbol{S_{0}}}{\sqrt{r_{2}^{2} + r_{3}^{2}} \boldsymbol{S_{0}}} = \frac{r_{2}}{\sqrt{r_{2}^{2}+r_{3}^{2}}}\\ \frac{\beta}{\sqrt{\alpha^{2} + \beta^{2}}} &=& \frac{r_{3} \boldsymbol{S_{0}}}{\sqrt{r_{2}^{2} + r_{3}^{2}} \boldsymbol{S_{0}}} = \frac{r_{3}}{\sqrt{r_{2}^{2}+r_{3}^{2}}}, \: \delta = r_{4} \boldsymbol{S_{0}}. \end{array} $$

Finally, let’s define two new parameters, *θ*_1_ and *θ*_2_ as $\theta _{1} = \frac {r_{1}}{\sqrt {r_{2}^{2}+r_{3}^{2}}}$ and $\theta _{2} \: = \: \tan ^{-1}\left (\frac {r_{3}}{r_{2}} \right)$. Using the above, we can rewrite ***UT***(*t*) as

$$\begin{array}{@{}rcl@{}} \boldsymbol{UT}(t) &=& \theta_{1} \: e^{r_{2} \boldsymbol{S_{0}} \: t} \left (\frac{r_{3}}{\sqrt{r_{2}^{2}+r_{3}^{2}}} \sin(r_{3} \boldsymbol{S_{0}} t - r_{4} \boldsymbol{S_{0}})\right. \\ &&\left. + \frac{r_{2}}{\sqrt{r_{2}^{2}+r_{3}^{2}}} \cos(r_{3} \boldsymbol{S_{0}} t - r_{4} \boldsymbol{S_{0}}) \right)\\ &&+ \theta_{1}\! \left(\! \frac{r_{3}}{\sqrt{r_{2}^{2}+r_{3}^{2}}} \sin(r_{4} \boldsymbol{S_{0}}) \!- \frac{r_{2}}{\sqrt{r_{2}^{2}+r_{3}^{2}}} \cos(r_{4} \boldsymbol{S_{0}})\! \right) \end{array} $$

Using a standard reference triangle for the phase angle *θ*_2_, we see $\cos (\theta _{2}) = \frac {r_{2}}{\sqrt {r_{2}^{2}+r_{3}^{2}}}$ and $\sin (\theta _{2}) = \frac {r_{3}}{\sqrt {r_{2}^{2}+r_{3}^{2}}}$. We can then rewrite ***UT***(*t*) again as

$$\begin{array}{@{}rcl@{}} \boldsymbol{UT}(t) &=& \theta_{1} \: e^{r_{2} \boldsymbol{S_{0}} \: t} (\sin(\theta_{2}) \sin(r_{3} \boldsymbol{S_{0}} t - r_{4} \boldsymbol{S_{0}})\\ &&+ \cos(\theta_{2}) \cos(r_{3} \boldsymbol{S_{0}} t - r_{4} \boldsymbol{S_{0}}))\\ &&+ \theta_{1} \left(\sin(\theta_{2}) \sin(r_{4} \boldsymbol{S_{0}}) - \cos(\theta_{2}) \cos(r_{4} \boldsymbol{S_{0}}) \right) \end{array} $$

and using standard trigonometric identities, we then have

$${} \boldsymbol{UT}(t) = \theta_{1} \: e^{r_{2} \boldsymbol{S_{0}} \: t} \cos(r_{3} \boldsymbol{S_{0}} t - r_{4} \boldsymbol{S_{0}} - \theta_{2}) - \theta_{1} \cos(r_{4} \boldsymbol{S_{0}}+\theta_{2}) $$

### The ***AT*** calculation

Recall, as showing in as shown in “Integration details”, we know

$$\begin{array}{@{}rcl@{}} \boldsymbol{AT}(t) &=& \int_{0}^{t} \boldsymbol{a}(s) ds = - \int_{0}^{t} \: R e^{-\alpha t} \sin(\beta s - \delta)\\ &=& \!-\frac{R}{\left(\alpha^{2} + \beta^{2}\right)} e^{\alpha t} \!\left (-\beta \cos(\beta t - \delta) + \alpha \sin(\beta t -\delta) \right)\\ && - \frac{R}{\alpha^{2}+\beta^{2}} \left(\beta \cos(\delta) + \alpha \sin(\delta) \right) \end{array} $$

Another standard integration by parts similar to what was done in “Integration details” allows us to find ***AT***. We note the same comment on the dependence of *R*, *α*, *β* and *δ* on ***S***_***0***_ holds still. Now using these values and the terms *Q*_1_ and *Q*_2_, we we can rewrite ***AT***(*t*) as follows:

$$\begin{array}{@{}rcl@{}} \boldsymbol{AT}(t) \,=\, &-&\!\frac{R}{\left(\alpha^{2} + \beta^{2}\right)} e^{\alpha t} \!\left (-\beta \cos(\beta t - \delta) + \alpha \sin(\beta t -\delta) \right)\\ &-& \frac{R}{\alpha^{2}+\beta^{2}} \left(\beta \cos(\delta) + \alpha \sin(\delta) \right) \end{array} $$

Now using the simplifications we obtained for *α* and *β* in terms of *r*_2_ and *r*_3_, we can rewrite this complicated expression as

$$\begin{array}{@{}rcl@{}} \boldsymbol{AT}(t) =&&-\theta_{1} e^{r_{2} \boldsymbol{S_{0}} t} \left (- \frac{r_{3}}{\sqrt{r_{2}^{2}+r_{3}^{2}}} \cos\left(r_{3} \boldsymbol{S_{0}} t - r_{4} \boldsymbol{S_{0}}\right)\right.\\ &&\left.+ \frac{r_{2}}{\sqrt{r_{2}^{2}+r_{3}^{2}}} \sin\left(r_{3} \boldsymbol{S_{0}} t - r_{4} \boldsymbol{S_{0}}\right) \right)\\ &&- \theta_{1}\!\left(\!\frac{r_{3}}{\sqrt{r_{2}^{2}+r_{3}^{2}}} \cos(r_{4} \boldsymbol{S_{0}}) \,+\, \frac{r_{2}}{\sqrt{r_{2}^{2}+r_{3}^{2}}} \sin(r_{4} \boldsymbol{S_{0}})\!\!\right) \end{array} $$

Next, using the phase shift *θ*_2_, we have

$$\begin{array}{@{}rcl@{}} \boldsymbol{AT}(t) = &&-\theta_{1} \: e^{r_{2} \boldsymbol{S_{0}} t}(- \sin(\theta_{2}) \cos(r_{3} \boldsymbol{S_{0}} t - r_{4} \boldsymbol{S_{0}})\\ &&+ \cos(\theta_{2}) \sin(r_{3} \boldsymbol{S_{0}} t - r_{4} \boldsymbol{S_{0}}))\\ &&- \theta_{1} \: \left(\sin(\theta_{2}) \cos(r_{4} \boldsymbol{S_{0}}) + \cos(\theta_{2}) \sin(r_{4} \boldsymbol{S_{0}}) \right) \end{array} $$

This then leads to our final form

$$\begin{array}{@{}rcl@{}} \boldsymbol{AT}(t) &\,=\,& -\theta_{1} \: e^{r_{2} \boldsymbol{S_{0}} t} \: \sin(r_{3} \boldsymbol{S_{0}} t \,-\, r_{4} \boldsymbol{S_{0}} \,-\, \theta_{2}) \,-\, \theta_{1} \sin(r_{4} \boldsymbol{S_{0}}\,+\,\theta_{2}) \end{array} $$

## Building the health model

Recall the health model is

$$\begin{array}{@{}rcl@{}} \boldsymbol{H}(t) \,=\, \boldsymbol{T} &\,-\,& \Lambda \boldsymbol{S_{0}} \,-\, H_{1\boldsymbol{i}}^{o} \: \boldsymbol{IT}(t) \,-\,\left(H_{1\boldsymbol{u}}^{o} + H_{2\boldsymbol{u}}^{o} + H_{3\boldsymbol{u}}^{o} \right) \: \boldsymbol{UT}(t)\\ &-&\left(H_{1\boldsymbol{a}}^{o} + H_{2\boldsymbol{a}}^{o} + H_{3\boldsymbol{a}}^{o} \right) \: \boldsymbol{AT}(t) \end{array} $$

Let $c_{u} = H_{1\boldsymbol {u}}^{o} + H_{2\boldsymbol {u}}^{o} + H_{3\boldsymbol {u}}^{o}$ and $c_{a} = H_{1\boldsymbol {a}}^{o} + H_{2\boldsymbol {a}}^{o} + H_{3\boldsymbol {a}}^{o}$. Then we have

$$\begin{array}{@{}rcl@{}} \boldsymbol{H}(t) &=& \boldsymbol{T} - \Lambda \: \boldsymbol{S_{0}} - H_{1\boldsymbol{i}}^{o} \: \boldsymbol{IT}(t) - c_{u} \: \boldsymbol{UT}(t) - c_{a} \: \boldsymbol{AT}(t) \end{array} $$

Now plug what we have found for our integrations. We have

$$\begin{array}{@{}rcl@{}} \boldsymbol{H}(t) = \boldsymbol{T} &-& \Lambda \boldsymbol{S_{0}} - H_{1\boldsymbol{i}}^{o} \frac{A}{G_{1\boldsymbol{i}}^{o}} \left(1 - e^{-G_{1\boldsymbol{i}}^{o} t} \right)\\ &-& c_{u} \: \left\{ \theta_{1} \: e^{r_{2} \boldsymbol{S_{0}} \: t} \cos(r_{3} \boldsymbol{S_{0}} t - r_{4} \boldsymbol{S_{0}} - \theta_{2})\right.\\ &&~~~~~~\left.- \theta_{1} \cos(\theta_{2}) \cos(r_{4} \boldsymbol{S_{0}}+\theta_{2}) {\vphantom{\theta_{1} \: e^{r_{2} \boldsymbol{S_{0}} \: t} \cos(r_{3} \boldsymbol{S_{0}} t - r_{4} \boldsymbol{S_{0}} - \theta_{2})}}\right\} \\ &-& c_{a} \: \left\{ -\theta_{1} \: e^{r_{2} \boldsymbol{S_{0}} t} \: \sin(r_{3} \boldsymbol{S_{0}} t - r_{4} \boldsymbol{S_{0}} - \theta_{2})\right.\\ &&~~~~~~\left. - \theta_{1} \: \sin(r_{4} \boldsymbol{S_{0}}+ \theta_{2}) {\vphantom{\theta_{1} \: e^{r_{2} \boldsymbol{S_{0}} \: t} \cos(r_{3} \boldsymbol{S_{0}} t - r_{4} \boldsymbol{S_{0}} - \theta_{2})}}\right\} \end{array} $$

Then we can rewrite as

$$\begin{array}{@{}rcl@{}} \boldsymbol{H}(t) = \boldsymbol{T} &-& \Lambda \boldsymbol{S_{0}} - H_{1\boldsymbol{i}}^{o} \frac{A}{G_{1\boldsymbol{i}}^{o}} \left(1 - e^{-G_{1\boldsymbol{i}}^{o} t} \right)\\ &-&\theta_{1} \: c_{u} \: \left\{ e^{r_{2} \boldsymbol{S_{0}} \: t} \cos(r_{3} \boldsymbol{S_{0}} t - r_{4} \boldsymbol{S_{0}} - \theta_{2})\right.\\ &&~~~~~~~~~~~\left.- \cos(r_{4} \boldsymbol{S_{0}}+\theta_{2}) {\vphantom{\theta_{1} \: e^{r_{2} \boldsymbol{S_{0}} \: t} \cos(r_{3} \boldsymbol{S_{0}} t - r_{4} \boldsymbol{S_{0}} - \theta_{2})}}\right\} \\ & +& \theta_{1} \: c_{a} \: \left\{ e^{r_{2} \boldsymbol{S_{0}} t} \: \sin(r_{3} \boldsymbol{S_{0}} t - r_{4} \boldsymbol{S_{0}} - \theta_{2})\right.\\ &&~~~~~~~~~~~\left. + \sin(r_{4} \boldsymbol{S_{0}}+ \theta_{2}) {\vphantom{\theta_{1} \: e^{r_{2} \boldsymbol{S_{0}} \: t} \cos(r_{3} \boldsymbol{S_{0}} t - r_{4} \boldsymbol{S_{0}} - \theta_{2})}}\right\} \end{array} $$

Now put the $\phantom {\dot {i}\!}e^{r_{2} \boldsymbol {S_{0}} t}$ together. We find

$$\begin{array}{@{}rcl@{}} \boldsymbol{H}(t) = \boldsymbol{T} &-& \Lambda \boldsymbol{S_{0}} - H_{1\boldsymbol{i}}^{o} \frac{A}{G_{1\boldsymbol{i}}^{o}} \left(1 - e^{-G_{1\boldsymbol{i}}^{o} t} \right)\\ & +& \theta_{1} \: \left(c_{u} \: \cos\left(r_{4} \boldsymbol{S_{0}}+\theta_{2}\right) \: + \: c_{a} \: \sin\left(r_{4} \boldsymbol{S_{0}} - \theta_{2}\right) \right) \\ &-&\theta_{1} \: e^{r_{2} \boldsymbol{S_{0}} t} \: \left(c_{u} \: \cos(r_{3} \boldsymbol{S_{0}} t - r_{4} \boldsymbol{S_{0}} - \theta_{2}\right)\\ &-& \: c_{a} \: \sin(r_{3} \boldsymbol{S_{0}} t - r_{4} \boldsymbol{S_{0}} - \theta_{2})) \end{array} $$

Let’s simplify some more using another phase shift. Define the phase angle $\theta _{3} = \tan ^{-1}\left (\frac {c_{u}}{c_{a}} \right)$; then, we can rewrite the health like this.

$$\begin{array}{@{}rcl@{}} \boldsymbol{H}(t) = \boldsymbol{T} &-& \Lambda \boldsymbol{S_{0}} - H_{1\boldsymbol{i}}^{o} \frac{A}{G_{1\boldsymbol{i}}^{o}} \left(1 - e^{-G_{1\boldsymbol{i}}^{o} t} \right)\\ &-\!& \theta_{1} \: e^{r_{2} \boldsymbol{S_{0}} t} \!\sqrt{c_{u}^{2} + c_{a}^{2}}\! \left(\! \frac{c_{u}}{\sqrt{c_{u}^{2} + c_{a}^{2}}} \cos(r_{3} \boldsymbol{S_{0}} t - r_{4} \boldsymbol{S_{0}} - \theta_{2})\right.\\ &&\left.- {c_{a}}{\sqrt{c_{u}^{2} + c_{a}^{2}}} \sin\left(r_{3} \boldsymbol{S_{0}} t - r_{4} \boldsymbol{S_{0}} - \theta_{2}\right) \right) \\ & +& \theta_{1} \sqrt{c_{u}^{2} + c_{a}^{2}} \: \left(\frac{c_{u}}{\sqrt{c_{u}^{2} + c_{a}^{2}}} \cos(r_{4} \boldsymbol{S_{0}}+\theta_{2})\right.\\ &&\left. + \frac{c_{a}}{\sqrt{c_{u}^{2} + c_{a}^{2}}} \: \sin\left(r_{4} \boldsymbol{S_{0}} - \theta_{2}\right) \right) \end{array} $$

This can be recast as

$$\begin{array}{@{}rcl@{}} \boldsymbol{H}(t) = \boldsymbol{T} &-& \Lambda \boldsymbol{S_{0}} - H_{1\boldsymbol{i}}^{o} \frac{A}{G_{1\boldsymbol{i}}^{o}} \left(1 - e^{-G_{1\boldsymbol{i}}^{o} t} \right)\\ &-& \theta_{1} \: e^{r_{2} \boldsymbol{S_{0}} t} \sqrt{c_{u}^{2} + c_{a}^{2}} \: (\cos(\theta_{3}) \: \cos\left(r_{3} \boldsymbol{S_{0}} t - r_{4} \boldsymbol{S_{0}} - \theta_{2}\right)\\ &-&\sin(\theta_{3}) \: \sin\left(r_{3} \boldsymbol{S_{0}} t - r_{4} \boldsymbol{S_{0}} - \theta_{2}\right)) \\ & +& \theta_{1} \sqrt{c_{u}^{2} + c_{a}^{2}} \: (\cos(\theta_{3}) \: \cos(r_{4} \boldsymbol{S_{0}}+\theta_{2})\\ &+& \sin(\theta_{3}) \: \sin(r_{4} \boldsymbol{S_{0}} - \theta_{2}))\\ =\boldsymbol{T} &-& \Lambda \boldsymbol{S_{0}} - H_{1\boldsymbol{i}}^{o} \frac{A}{G_{1\boldsymbol{i}}^{o}} \left(1 - e^{-G_{1\boldsymbol{i}}^{o} t} \right)\\ &-& \theta_{1} \: e^{r_{2} \boldsymbol{S_{0}} t} \sqrt{c_{u}^{2} + c_{a}^{2}} \: \cos(r_{3} \boldsymbol{S_{0}} t - r_{4} \boldsymbol{S_{0}} - \theta_{2} + \theta_{3})\\ & +& \theta_{1} \sqrt{c_{u}^{2} + c_{a}^{2}} \: \cos(r_{4} \boldsymbol{S_{0}} - \theta_{2} -\theta_{3}) \end{array} $$

Next, we can combine the ratio $H_{1\boldsymbol {i}}^{o} \frac {A}{G_{1\boldsymbol {i}}^{\boldsymbol {S_{0}}}}$ into the new parameter $\zeta _{1}^{\boldsymbol {S_{0}}}$ and rewrite $G_{1\boldsymbol {i}}^{\boldsymbol {S_{0}}}$ as $\zeta _{2}^{\boldsymbol {S_{0}}}$ to give

$$\begin{array}{@{}rcl@{}} \boldsymbol{H}(t) = \boldsymbol{T} &-& \Lambda \boldsymbol{S_{0}} - \zeta_{1}^{\boldsymbol{S_{0}}} \left(1 - e^{- \zeta_{2}^{\boldsymbol{S_{0}}} t} \right)\\ & - &\theta_{1} e^{r_{2} \boldsymbol{S_{0}} t} \sqrt{c_{u}^{2} + c_{a}^{2}} \: \cos(r_{3} \boldsymbol{S_{0}} t \!- r_{4} \boldsymbol{S_{0}} - \theta_{2} + \theta_{3})\\ & +& \theta_{1} \sqrt{c_{u}^{2} + c_{a}^{2}} \: \cos(r_{4} \boldsymbol{S_{0}} - \theta_{2} -\theta_{3}) \end{array} $$

Finally, let $s_{1} = \theta _{1} \: \sqrt {c_{u}^{2} + c_{a}^{2}}$. Then, we have the last form of the health estimate:

1 $$\begin{array}{@{}rcl@{}} \boldsymbol{H}(t) = \boldsymbol{T} &-& \Lambda \boldsymbol{S_{0}} - \zeta_{1}^{\boldsymbol{S_{0}}} \left(1 - e^{- \zeta_{2}^{\boldsymbol{S_{0}}} t} \right)\notag\\ & - &s_{1} e^{r_{2} \boldsymbol{S_{0}} t} \: \cos(r_{3} \boldsymbol{S_{0}} t - r_{4} \boldsymbol{S_{0}} - \theta_{2} + \theta_{3}) \end{array} $$

2 $$\begin{array}{@{}rcl@{}} & +& s_{1} \cos(r_{4} \boldsymbol{S_{0}} - \theta_{2} -\theta_{3})  \end{array} $$

We could also assume the terms $\zeta _{1}^{\boldsymbol {S_{0}}}$ and $\zeta _{2}^{\boldsymbol {S_{0}}}$ are proportional to ***S***_***0***_. We would model this by implying $\zeta _{1}^{\boldsymbol {S_{0}}} = r_{5} \boldsymbol {S_{0}}$ and $\zeta _{2}^{\boldsymbol {S_{0}}} = r_{6} \boldsymbol {S_{0}}$. We then find

3 $$\begin{array}{@{}rcl@{}} \boldsymbol{H}(t) = \boldsymbol{T} &-& \Lambda \boldsymbol{S_{0}} - r_{5} \: \boldsymbol{S_{0}} \left(1 - e^{- r_{6} \boldsymbol{S_{0}} t} \right)\notag\\ &-& s_{1} e^{r_{2} \boldsymbol{S_{0}} t} \: \cos(r_{3} \boldsymbol{S_{0}} t - r_{4} \boldsymbol{S_{0}} - \theta_{2} + \theta_{3}) \end{array} $$

4 $$\begin{array}{@{}rcl@{}} &+& s_{1} \cos(r_{4} \boldsymbol{S_{0}} - \theta_{2} -\theta_{3})  \end{array} $$

These parameters depend in complex ways on the initial virus dose ***S***_***0***_ and it is very difficult to tease out the details. The only data we have to help fit this model is the survival data, so the next step of seeing how this model of health gives rise to the experimental survival data requires additional analysis.

## Results and discussions

**Fig. 2:**
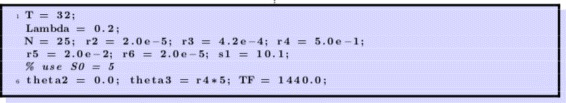
Set the parameters

We are going to graph Eq. 4 for various values of the parameters. First, we set the parameters as showin in Fig. 2.

**Fig. 3:**
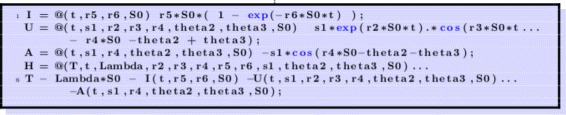
Define needed functions

Then, we define some auxiliary functions in Fig. 3.

**Fig. 4:**
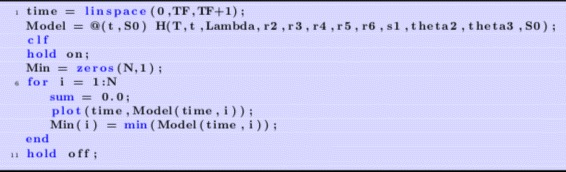
Generating survival plots

**Fig. 5:**
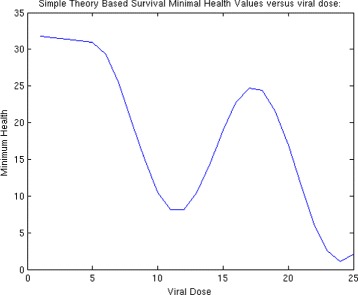
Minimum heath versus viral dose

**Fig. 6:**
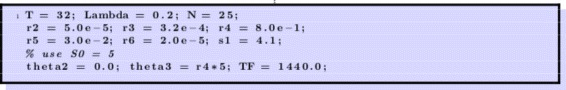
Set the parameters for another run

**Fig. 7:**
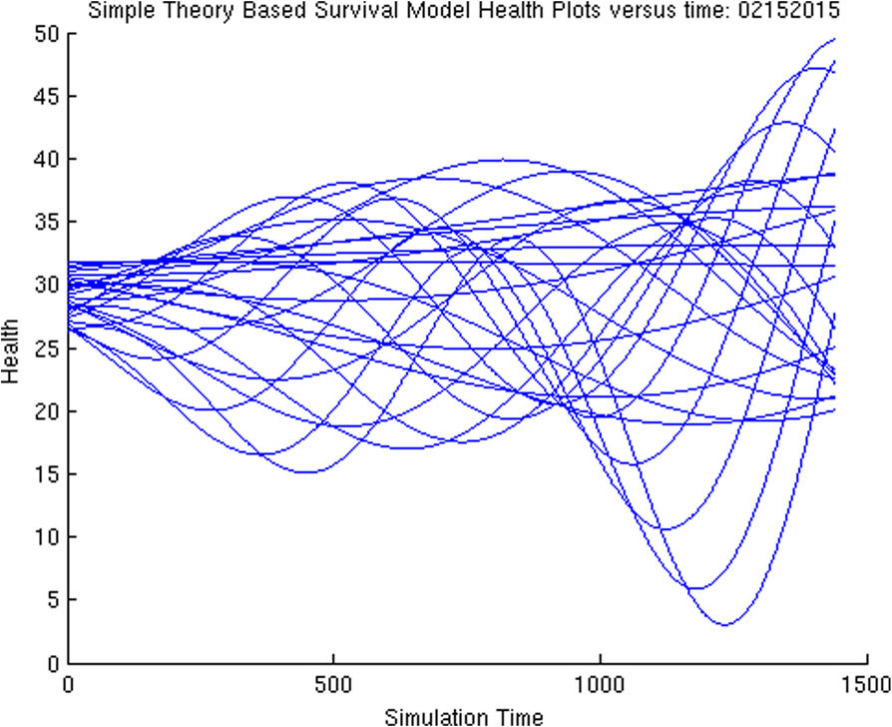
Heath plots versus time for many viral doses

**Fig. 8:**
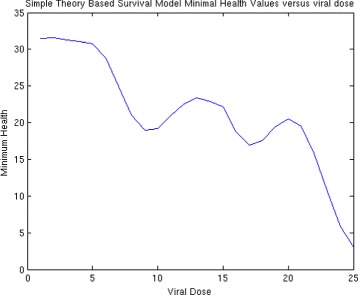
Minimum heath versus viral dose

**Fig. 9:**
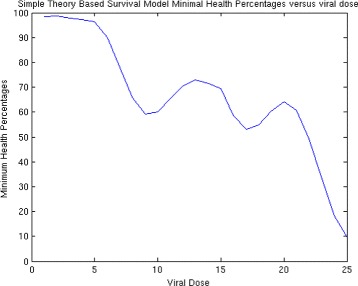
Minimal heath percentage versus viral dose

Then, we generate a plot of health versus time for a range of choices of ***S***_***0***_, here represented by **S0** as shown in Fig. 4. For each plot, we find the minimum value and store it in the variable **Min**. We can then plot **Min** versus initial viral dose **S0** and see if the plot looks like a traditional WNV survival curve. We then place all of this code into a MatLab function and use it in the usual way to generate the plots. For these choice of parameters, we can plot the minimal health curve which is shown in Fig. 5. Another choice of parameters, leads to an even better minimum health curve which captures the essence of the WNV survival plot. The parameter choices for this run are shown in Fig. 6. For these parameters values, all of the health plots versus time can be seen in Fig. 7 and show much oscillation. The corresponding minimal health curve is then shown in Fig. 8. We can make this look more like the real survival data by scaling the minimal health values and plotting them as a percentage. When we do this, we find a plot that is a bit easier to compare to the real data. It is not perfect, of course, but it is very interesting that we are capturing the essential quality of the data using our theoretical model. The percentage minimal values are shown in Fig. 9. From these experiments, it is clear what is happening. The model

$$\begin{array}{@{}rcl@{}} \boldsymbol{H}(t) = \boldsymbol{T} &-& \Lambda \boldsymbol{S_{0}} - r_{5} \: \boldsymbol{S_{0}} \left(1 - e^{- r_{6} \boldsymbol{S_{0}} t} \right)\\ & -& s_{1} e^{r_{2} \boldsymbol{S_{0}} t} \: \cos(r_{3} \boldsymbol{S_{0}} t - r_{4} \boldsymbol{S_{0}} - \theta_{2} + \theta_{3})\\ &+& s_{1} \cos(r_{4} \boldsymbol{S_{0}} - \theta_{2} -\theta_{3}) \end{array} $$

can be written in terms of **decay** and **push - pull** terms as follows:

$${} {\begin{aligned} &~~~~~~~~~~~~~~~~~~~~~~~~~~~~~~~~~~~~~~~~~~~~~~~~~~~~~~~~~~~~~~~~~~~~- \Lambda \boldsymbol{S_{0}} = \mathbf{decay }\\ &~~~~~~~~~~~~~~~~~~~~~~~~~~~~~ ~~~~~~~~~~~~~~~- r_{5} \: \boldsymbol{S_{0}} \left(1 - e^{- r_{6} \boldsymbol{S_{0}} t} \right) = \mathbf{decay}\\ &- s_{1} e^{r_{2} \boldsymbol{S_{0}} t} \cos(r_{3} \boldsymbol{S_{0}} t - r_{4} \boldsymbol{S_{0}} - \theta_{2} + \theta_{3})\! + s_{1} \cos(r_{4} \boldsymbol{S_{0}} \!- \theta_{2} -\theta_{3})\\ &~~~~~~~~~~~~~~~~~~~~~~~~~~~~~~~~~~~~~~~~~~~~~~~~~~~~~~~~~~~~~~~~~~~~~~~~~~~~~~~~= \mathbf{push - pull } \end{aligned}} $$

Thus, we have ***H***(*t*) always decreases unless the **push - pull** terms counteract that decay. Hence, what is important is the term

$$\begin{array}{@{}rcl@{}} \boldsymbol{\Delta}(t) &=& - s_{1} e^{r_{2} \boldsymbol{S_{0}} t} \: \cos(r_{3} \boldsymbol{S_{0}} t - r_{4} \boldsymbol{S_{0}} - \theta_{2} + \theta_{3})\\ &&+ s_{1} \cos(r_{4} \boldsymbol{S_{0}} - \theta_{2} -\theta_{3}) \end{array} $$

can oscillate as viral load increases. To do this, it is important for the two terms in ***Δ***(*t*) to be out of phase. Hence, roughly speaking cos(*r*_3_ ***S***_***0***_ *t*−*r*_4_ ***S***_***0***_−*θ*_2_+*θ*_3_) must be sometimes negative when cos(*r*_4_ ***S***_***0***_−*θ*_2_−*θ*_3_) is positive. This allows for an increase in health of approximately *ξ* *s*_1_ where *ξ* is the difference between the two terms. This is possible when the two cos arguments are out of phase by about *π* radians. Note it is also important that the exponential term $e^{r_{2} \boldsymbol {S_{0}} t}\phantom {\dot {i}\!}$ allows growth. The interaction dynamics are determined by

$$\begin{array}{@{}rcl@{}} \left[\begin{array}{cc} G_{2\boldsymbol{u}}^{\boldsymbol{S_{0}}} & G_{2\boldsymbol{a}}^{\boldsymbol{S_{0}}}\\ G_{3\boldsymbol{u}}^{\boldsymbol{S_{0}}} & G_{3\boldsymbol{a}}^{\boldsymbol{S_{0}}} \end{array}\right] = \left[\begin{array}{cc} \alpha & - \beta\\ \beta & \alpha \end{array}\right] \end{array} $$

and we have argued that the appropriate algebraic signs for this coefficient matrix $\boldsymbol {\mathcal {M}}$ are

$$\begin{array}{@{}rcl@{}} \boldsymbol{\mathcal{M}} &=&\left[\begin{array}{ll} + & - \\ + & + \end{array}\right] \end{array} $$

We can have complex eigenvalues and hence oscillating behavior if the signs were

$$\begin{array}{@{}rcl@{}} \boldsymbol{\mathcal{M}} &=&\left[\begin{array}{ll} - & + \\ - & - \end{array}\right] \end{array} $$

but then the real part of the eigenvalues would be negative and we would have to model the exponential term as $e^{-r_{2} \boldsymbol {S_{0}} t}\phantom {\dot {i}\!}$. The induced oscillations would then be damped and the potential for the WNV survival curve would vanish.

## Collateral damage

We should also see oscillations in the collateral damage. Recall the collateral damage population is given by

$$\begin{array}{@{}rcl@{}} \boldsymbol{C}(t) =& \boldsymbol{C_{0}} &+H_{1\boldsymbol{i}}^{o} \: \left (\int_{0}^{t} \boldsymbol{i}(s) ds \right)\\ &+& H_{1\boldsymbol{u}}^{o} \: \left (q_{1} \boldsymbol{S_{0}} + \int_{0}^{t} \boldsymbol{u}(s) ds \right)\\ &+&H_{1\boldsymbol{a}}^{o} \: \left ((1-p_{0}) \boldsymbol{S_{0}} + \int_{0}^{t} \boldsymbol{a}(s) ds \right)\\ =& \boldsymbol{C_{0}} &+ H_{1\boldsymbol{i}}^{o} \: \boldsymbol{IT}(t) + H_{1\boldsymbol{u}}^{o}\: \left (q_{1} \boldsymbol{S_{0}} + \boldsymbol{UT}(t) \right)\\ &+& H_{1\boldsymbol{a}}^{o} \: \left ((1-p_{0}) \boldsymbol{S_{0}} + \boldsymbol{AT}(t) \right) \end{array} $$

We can then substitute for ***IT***(*t*), ***UT***(*t*) and ***AT***(*t*) and obtain

$$\begin{array}{@{}rcl@{}} \boldsymbol{C}(t) = \boldsymbol{C_{0}} &+&r_{5} \boldsymbol{S_{0}} \left(1 - e^{- r_{6}\boldsymbol{S_{0}} t} \right)\\ & +& H_{1\boldsymbol{u}}^{o} \! \left(q_{1} \boldsymbol{S_{0}} + \theta_{1} \: e^{r_{2} \boldsymbol{S_{0}} \: t} \cos(r_{3} \boldsymbol{S_{0}} t \!- r_{4} \boldsymbol{S_{0}} - \theta_{2})\right.\\ &&\left.- \theta_{1} \cos(r_{4} \boldsymbol{S_{0}}+\theta_{2}) \right)\\ & +& H_{1\boldsymbol{a}}^{o}\! \left(\!(1-p_{0}) \boldsymbol{S_{0}} \!-\theta_{1} \: e^{r_{2} \boldsymbol{S_{0}} t} \: \sin(r_{3} \boldsymbol{S_{0}} t - r_{4} \boldsymbol{S_{0}} - \theta_{2})\right.\\ &&\left. - \theta_{1} \: \sin(r_{4} \boldsymbol{S_{0}}+ \theta_{2}) \right) \end{array} $$

Now, collect terms as we did in our earlier simplifications. We rewrite as

$$\begin{array}{@{}rcl@{}} \boldsymbol{C}(t) = \boldsymbol{C_{0}} &\,+\,&r_{5} \boldsymbol{S_{0}} \!\left(1 - e^{- r_{6}\boldsymbol{S_{0}} t} \right) \,+\, \left(H_{1\boldsymbol{a}}^{o} \: (1\!-p_{0}) + H_{1\boldsymbol{u}}^{o} \: q_{1}\right) \: \boldsymbol{S_{0}}\\ &+& \theta_{1} \: e^{r_{2} \boldsymbol{S_{0}} t} \left(H_{1\boldsymbol{u}}^{o}\:\cos(r_{3} \boldsymbol{S_{0}} t - r_{4} \boldsymbol{S_{0}} - \theta_{2})\right.\\ &&\left. - H_{1\boldsymbol{a}}^{o}\: \sin(r_{3} \boldsymbol{S_{0}} t - r_{4} \boldsymbol{S_{0}} - \theta_{2}) \right)\\ & -& \theta_{1} \left(H_{1\boldsymbol{u}}^{o}\: \cos(r_{4} \boldsymbol{S_{0}}+\theta_{2}) +H_{1\boldsymbol{a}}^{o}\: \sin(r_{4} \boldsymbol{S_{0}}+ \theta_{2}) \right) \end{array} $$

We can also introduce an additional phase shift, *ϕ*, as follows. It will be different from the phase shift

$$\begin{array}{@{}rcl@{}} \theta_{3} &=& \tan^{-1}\left(\frac{c_{u}}{c_{a}}\right) = \tan^{-1} \left(\frac{H_{1\boldsymbol{u}}^{o}+H_{2\boldsymbol{u}}^{o}+H_{3\boldsymbol{u}}^{o}} {H_{1\boldsymbol{a}}^{o}+H_{2\boldsymbol{a}}^{o}+H_{3\boldsymbol{a}}^{o}} \right) \end{array} $$

as here we only use the *H*_1_ partials: $\phi =\tan ^{-1} \left (\frac {H_{1\boldsymbol {a}}^{o}}{H_{1\boldsymbol {u}}^{o}} \right)$. We rewrite as

$${} {\begin{aligned} \boldsymbol{C}(t) = \boldsymbol{C_{0}} &+r_{5} \boldsymbol{S_{0}} \left(1 - e^{- r_{6}\boldsymbol{S_{0}} t} \right) + \left(H_{1\boldsymbol{a}}^{o} \: (1-p_{0}) + H_{1\boldsymbol{u}}^{o} \: q_{1}\right) \: \boldsymbol{S_{0}}\\ &+\!\theta_{1} \sqrt{\left(H_{1\boldsymbol{u}}^{o}\right)^{2}+\left(H_{1\boldsymbol{a}}^{o}\right)^{2}} e^{r_{2} \boldsymbol{S_{0}} t} \!(\cos(\phi) \cos(r_{3} \boldsymbol{S_{0}} t \,-\, r_{4} \boldsymbol{S_{0}} \,-\, \theta_{2})\\ &- \sin(\phi) \sin(r_{3} \boldsymbol{S_{0}} t - r_{4} \boldsymbol{S_{0}} - \theta_{2}))\\ & - \theta_{1}\: \sqrt{\left(H_{1\boldsymbol{u}}^{o}\right)^{2}+\left(H_{1\boldsymbol{a}}^{o}\right)^{2}}\: (\cos(\phi) \: \cos(r_{4} \boldsymbol{S_{0}}+\theta_{2})\\ &+\sin(\phi) \: \sin(r_{4} \boldsymbol{S_{0}}+ \theta_{2})) \end{aligned}} $$

We can then use the the usual cos laws of addition and subtraction of angles to repackage this as

$$\begin{array}{@{}rcl@{}} \boldsymbol{C}(t) = \boldsymbol{C_{0}} &+& r_{5} \boldsymbol{S_{0}} \left(1 - e^{- r_{6}\boldsymbol{S_{0}} t} \right) + \left(H_{1\boldsymbol{a}}^{o} \: (1-p_{0}) + H_{1\boldsymbol{u}}^{o} \: q_{1}\right) \: \boldsymbol{S_{0}}\\ &+& \theta_{1} \sqrt{\left(H_{1\boldsymbol{u}}^{o}\right)^{2}\,+\,(H_{1\boldsymbol{a}}^{o})^{2}} e^{r_{2} \boldsymbol{S_{0}} t} \cos(r_{3} \boldsymbol{S_{0}} t\! - r_{4} \boldsymbol{S_{0}} - \theta_{2}+\phi) \\ & -& \theta_{1}\: \sqrt{(H_{1\boldsymbol{u}}^{o})^{2}+(H_{1\boldsymbol{a}}^{o})^{2}}\: \cos(r_{4} \boldsymbol{S_{0}}+\theta_{2}-\phi) \end{array} $$

Now define $s_{2} = \theta _{1}\: \sqrt {\left (H_{1\boldsymbol {u}}^{o}\right)^{2}+\left (H_{1\boldsymbol {a}}^{o}\right)^{2}}$ and rewrite as

$$\begin{array}{@{}rcl@{}} \boldsymbol{C}(t) = \boldsymbol{C_{0}} &+& r_{5} \boldsymbol{S_{0}} \left(1 - e^{- r_{6}\boldsymbol{S_{0}} t} \right) + \left(H_{1\boldsymbol{a}}^{o} \: (1-p_{0}) + H_{1\boldsymbol{u}}^{o} \: q_{1}\right) \: \boldsymbol{S_{0}}\\ &+& s_{2} \:e^{r_{2} \boldsymbol{S_{0}} t} \cos(r_{3} \boldsymbol{S_{0}} t - r_{4} \boldsymbol{S_{0}} - \theta_{2}+\phi)\\ &-& s_{2} \: \cos(r_{4} \boldsymbol{S_{0}}+\theta_{2}-\phi) \end{array} $$

Since collateral damage is initially zero, we have as our final form

$$\begin{array}{@{}rcl@{}} \boldsymbol{C}(t) &=& r_{5} \boldsymbol{S_{0}} \left(1 - e^{- r_{6}\boldsymbol{S_{0}} t} \right) + \left(H_{1\boldsymbol{a}}^{o} \: (1-p_{0}) + H_{1\boldsymbol{u}}^{o} \: q_{1}\right) \: \boldsymbol{S_{0}}\\ && + s_{2} \:e^{r_{2} \boldsymbol{S_{0}} t} \cos(r_{3} \boldsymbol{S_{0}} t - r_{4} \boldsymbol{S_{0}} - \theta_{2}+\phi)\\ &&- s_{2} \: \cos(r_{4} \boldsymbol{S_{0}}+\theta_{2}-\phi) \end{array} $$

Previously, we used the simplification

$$\begin{array}{@{}rcl@{}} \Lambda &=& (p_{1} + p_{2}) p_{0} + \left(H_{1\boldsymbol{u}}^{o} + H_{2\boldsymbol{u}}^{o} + H_{3\boldsymbol{u}}^{o} \right) \: q_{1}\\ &+&\left(H_{1\boldsymbol{a}}^{o} + H_{2\boldsymbol{a}}^{o} + H_{3\boldsymbol{a}}^{o} \right) \: (1-p_{0}) \end{array} $$

This needs to be modified to

$$\begin{array}{@{}rcl@{}} \Lambda^{1} &=& H_{1\boldsymbol{u}}^{o} \: q_{1} +H_{1\boldsymbol{a}}^{o} \: (1-p_{0}). \end{array} $$

Hence, although we can use some of parameter choices from our health simulations, it is difficult to compare completely as *S*_2_, *Λ* ^1^ and *ϕ* are different. Our final collateral damage function is then

$$\begin{array}{@{}rcl@{}} \boldsymbol{C}(t) &=& \Lambda^{1} \boldsymbol{S_{0}} + r_{5} \boldsymbol{S_{0}} \left(1 - e^{- r_{6}\boldsymbol{S_{0}} t} \right) + s_{2} \:e^{r_{2} \boldsymbol{S_{0}} t} \cos(r_{3} \boldsymbol{S_{0}} t\\ &&- r_{4} \boldsymbol{S_{0}} - \theta_{2}+\phi) - s_{2} \: \cos(r_{4} \boldsymbol{S_{0}}+\theta_{2}-\phi) \end{array} $$

**Fig. 10:**
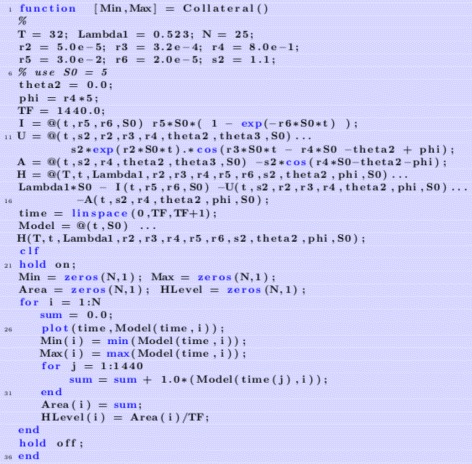
Collateral damage calculations

**Fig. 11:**
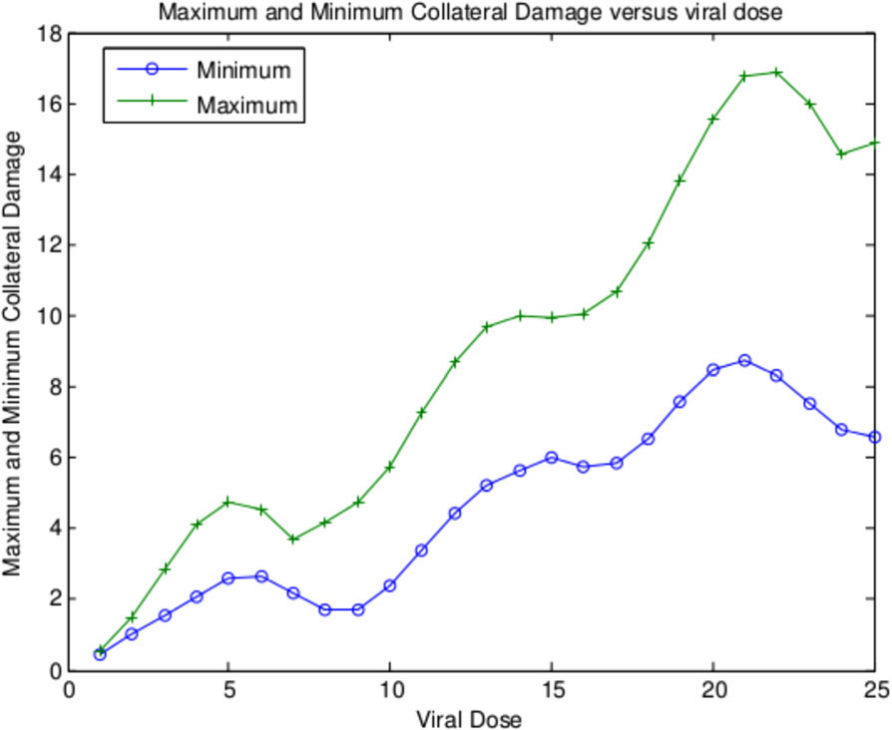
Collateral damage versus viral dose

We can easily run a quick simulation to see if our prediction that the collateral damage will have oscillations is true or not. We use the function **Collateral()** for this which is listed in Fig. 10. The parameter values we use are as similar as possible to the ones we used in generated the survival curves, although the values of *s*_2_, *Λ* ^1^ and *ϕ* are necessarily new choices. We ran the simulation with these parameter values and then plotted both the maximum and minimum collateral values versus the viral dose in Fig. 11. Note that there is variation in the collateral damage due to the nonlinear interactions between the upregulation, ***u***, and the free virus, ***a***.

## Conclusions

The presence of a form of self damage in our WNV infection model therefore appears to be a consequence of the nonlinear interactions in the ***i***, ***u*** and ***a*** model:

$$\begin{array}{@{}rcl@{}} \left[\begin{array}{l} \boldsymbol{i}^{\prime}\\ \boldsymbol{u}^{\prime}\\ \boldsymbol{d}^{\prime} \end{array}\right] \approx \left[\begin{array}{lll} G_{1\boldsymbol{i}}^{\boldsymbol{S_{0}}} & G_{1\boldsymbol{u}}^{\boldsymbol{S_{0}}} & G_{1\boldsymbol{a}}^{\boldsymbol{S_{0}}} \\ G_{2\boldsymbol{i}}^{\boldsymbol{S_{0}}} & G_{2\boldsymbol{u}}^{\boldsymbol{S_{0}}} & G_{2\boldsymbol{a}}^{\boldsymbol{S_{0}}} \\ G_{3\boldsymbol{i}}^{\boldsymbol{S_{0}}} & G_{3\boldsymbol{u}}^{\boldsymbol{S_{0}}} & G_{3\boldsymbol{a}}^{\boldsymbol{S_{0}}} \end{array}\right] \left[\begin{array}{l} \boldsymbol{i}\\ \boldsymbol{u}\\ \boldsymbol{a} \end{array}\right] \end{array} $$

Note, if the two populations ***D*** and ***C*** coincide, one signal is unnecessary - say *u* - and this model reduces to a two dimensional model

$$\begin{array}{@{}rcl@{}} \left[\begin{array}{l} \boldsymbol{i}^{\prime}\\ \boldsymbol{a}^{\prime} \end{array}\right] \approx \left[\begin{array}{ll} G_{1\boldsymbol{i}}^{\boldsymbol{S_{0}}} & G_{1\boldsymbol{a}}^{\boldsymbol{S_{0}}} \\ [3pt] G_{3\boldsymbol{i}}^{\boldsymbol{S_{0}}} & G_{3\boldsymbol{a}}^{\boldsymbol{S_{0}}} \end{array}\right] \left[\begin{array}{l} \boldsymbol{i}\\ \boldsymbol{a} \end{array}\right] \end{array} $$

and the chance of oscillation between the cellular population groups is lost. Hence, we can note some consequences and predictions due to our model.

- The crucial assumption here is that the viral infections effect on the host divides into two parts. For a WNV infection, these two cell populations are the dividing and nondividing infected cells, ***D*** and ***N***, respectively. We can envision other infectious agents or triggers that give rise to such a *split* response which then in principle could engender a similar collateral damage response which we interpret as an autoimmune reaction. So there is hope that this approach could perhaps give us insight into more general autoimmune responses. Note that Fig. 11 shows there is collateral damage that oscillates due to the infectious agent which here is WNV. It is clear that other triggering events, another virus or bacteria or even an environment toxin, could give rise to this behavior as well.
- Specific to the WNV model, we assume that $G_{2\boldsymbol {u}}^{\boldsymbol {S_{0}}} = +$, $G_{3\boldsymbol {u}}^{\boldsymbol {S_{0}}} = +$, $G_{2\boldsymbol {a}}^{\boldsymbol {S_{0}}} = -$, $G_{3\boldsymbol {a}}^{\boldsymbol {S_{0}}} = +$, which then says the coefficient matrix of the linearized upregulation and free virus model has the form
  $$\begin{array}{@{}rcl@{}} \left[\begin{array}{ll} G_{2\boldsymbol{u}}^{\boldsymbol{S_{0}}} & -G_{2\boldsymbol{a}}^{\boldsymbol{S_{0}}}\\ G_{3\boldsymbol{u}}^{\boldsymbol{S_{0}}} & G_{3\boldsymbol{a}}^{\boldsymbol{S_{0}}}\\ \end{array}\right] &=& \left[\begin{array}{ll} + & -\\ + & +\\ \end{array}\right] \end{array} $$
  This algebraic sign pattern itself can give rise to complex eigenvalues for the linearized nonlinear interaction model and we have not explored this more general problem. We have noted in our discussion in Section “Results and discussions” that if we did not have $G_{2\boldsymbol {u}}^{\boldsymbol {S_{0}}} = +$, we could still have oscillatory behavior but it would be damped and therefore it would not explain the data we see in the survival experiments. Here, we have posited **specific** relations that give rise to clearcut oscillations. We have assumed $G_{2\boldsymbol {u}}^{\boldsymbol {S_{0}}} = G_{3\boldsymbol {a}}^{\boldsymbol {S_{0}}}$ and $G_{3\boldsymbol {u}}^{\boldsymbol {S_{0}}} = -G_{2\boldsymbol {a}}^{\boldsymbol {S_{0}}}$ which gives rise to the characteristic coefficient matrix
  $$\begin{array}{@{}rcl@{}} \left[\begin{array}{cc} G_{2\boldsymbol{u}}^{\boldsymbol{S_{0}}} & -G_{3\boldsymbol{u}}^{\boldsymbol{S_{0}}}\\ G_{3\boldsymbol{u}}^{\boldsymbol{S_{0}}} & G_{2\boldsymbol{u}}^{\boldsymbol{S_{0}}} \end{array}\right] &=&\left[\begin{array}{cc} \alpha^{\boldsymbol{S_{0}}} & -\beta^{\boldsymbol{S_{0}}}\\ \beta^{\boldsymbol{S_{0}}} & \alpha^{\boldsymbol{S_{0}}} \end{array}\right] \end{array} $$
- The assumptions above then give rise to a general model of how the minimal health changes with varying initial virus dose, which appears to allow us to approximate the data we can measure.

The exact replication of the data found in the biological situation is unlikely to occur. Indeed, a standard dose response curve of survival does not usually repeat exactly from experiment to experiment, except in the form of it, even when using genetically identical animals. The ragged dose response of mortality seen in WNV infection is similarly subject to biological variability. Clearly, with no virus there will be 100% survival, and with a large amount of virus there will be 100% death, as expected in a standard dose response curve. In between these two doses, however, the response to infection is subject to probability, which affects the outcome (survival or death). Thus, if the experiment were undertaken several times it would show the ragged form on each occasion, but not exactly the same percentage survival at each dose used. This implies that small biological differences at the starting point of infection, albeit in genetically identical mice, may subtend a large range of endpoints. It is of interest to note that bypassing the early initiation of the adaptive immune response, by inoculating virus intracranially, that the standard graded dose response occurs with WNV. This is because the replication of the virus overtakes the animal before an effective immune response can be generated, emphasising the role of the immune system in generating this ragged survival curve [12].

## Conclusions

We have shown that we can build a reasonable model of how WNV infects a host’s cell in such a way that damage to the host can decrease, even though the inoculating viral dose increases.

## Methods

Our model is a macro one and we believe it provides insight as to how we can model more general auto-immune reactions. We propose that for an infectious agent or trigger to cause oscillations in health it is required that the trigger causes alterations in two distinct cell populations. Then, if the nonlinear interactions between these two populations satisfies the conditions for damped oscillatory response we have mentioned here, we should see oscillations in the host health. We consider this work essentially a theoretical model and as we explore what we can do to extend the results to more general auto-immune settings, we hope that we can generate greater mechanistic insight into auto-immune disease.
